# Tracking of Diversity and Evolution in the Brown Rot Fungi *Monilinia fructicola*, *Monilinia fructigena*, and *Monilinia laxa*

**DOI:** 10.3389/fmicb.2022.854852

**Published:** 2022-03-09

**Authors:** Rita Milvia De Miccolis Angelini, Lucia Landi, Celeste Raguseo, Stefania Pollastro, Francesco Faretra, Gianfranco Romanazzi

**Affiliations:** ^1^Department of Soil, Plant and Food Sciences, University of Bari Aldo Moro, Bari, Italy; ^2^Department of Agricultural, Food and Environmental Sciences, Marche Polytechnic University, Ancona, Italy

**Keywords:** biosynthetic gene clusters, effectors, phylogenetic analysis, repeat-induced point mutation, synteny, transposable elements

## Abstract

*Monilinia* species are among the most devastating fungi worldwide as they cause brown rot and blossom blight on fruit trees. To understand the molecular bases of their pathogenic lifestyles, we compared the newly assembled genomes of single strains of *Monilinia fructicola*, *M. fructigena* and *M. laxa*, with those of *Botrytis cinerea* and *Sclerotinia sclerotiorum*, as the closest species within *Sclerotiniaceae*. Phylogenomic analysis of orthologous proteins and syntenic investigation suggest that *M. laxa* is closer to *M. fructigena* than *M. fructicola*, and is closest to the other investigated *Sclerotiniaceae* species. This indicates that *M. laxa* was the earliest result of the speciation process. Distinct evolutionary profiles were observed for transposable elements (TEs). *M. fructicola* and *M. laxa* showed older bursts of TE insertions, which were affected (mainly in *M. fructicola*) by repeat-induced point (RIP) mutation gene silencing mechanisms. These suggested frequent occurrence of the sexual process in *M. fructicola*. More recent TE expansion linked with low RIP action was observed in *M. fructigena*, with very little in *S. sclerotiorum* and *B. cinerea*. The detection of active non-syntenic TEs is indicative of horizontal gene transfer and has resulted in alterations in specific gene functions. Analysis of candidate effectors, biosynthetic gene clusters for secondary metabolites and carbohydrate-active enzymes, indicated that *Monilinia* genus has multiple virulence mechanisms to infect host plants, including toxins, cell-death elicitor, putative virulence factors and cell-wall-degrading enzymes. Some species-specific pathogenic factors might explain differences in terms of host plant and organ preferences between *M. fructigena* and the other two *Monilinia* species.

## Introduction

*Monilinia fructicola* (G. Winter) Honey, *Monilinia fructigena* (Pers.) Honey and Monilinia *laxa* (Aderh. and Ruhland) Honey are the main causal agents of brown rot and blossom blight on fruit trees. They are Ascomycetes fungi that are included in the family *Sclerotiniaceae*, order *Helotiales*, and they are responsible for one of the most important and common diseases on stone and pome fruit in the field, as well as postharvest, causing heavy yield losses and reducing shelf-life (Petróczy et al., 2012; Martini and Mari, 2014).

*Monilinia laxa* was the prevalent species in California (United States) in the first half of the 20th century (Hewitt and Leach, 1939), and was first identified as the causal agent of brown rot on stone fruit in Europe (Byrde and Willets, 1977). Before the turn of the millennium, only *M. laxa* and *M. fructigena* were present in Europe (van Leeuwen et al., 2001). *M. laxa* is a quarantine pathogen in China and in some parts of North America (Martini and Mari, 2014).

*Monilinia fructigena* is more prevalent on pome fruit, while it has relatively low incidence on stone fruit (Martini and Mari, 2014). *M. fructigena* is widespread in Europe, Asia (e.g., Near East, Far East, India), northern Africa, and some parts of South America, and it is a quarantine pathogen in Canada, United States, Australia and New Zealand^1^.

*M. fructicola* was originally identified in North and South America, Australia, and Japan (Smith et al., 1997). In 2001, it was firstly reported in Europe on peach in France (Lichou et al., 2002), and in 2005 it was included in the A2 European and Mediterranean Plant Protection organization list of quarantine organisms (EPPO, 2021) due to the high yield losses reported on peach, apricot and nectarine (EFSA Panel on Plant Health [PLH], 2011).

Other *Monilinia* species include *Monilinia polystroma* (G. Leeuwen) L.M. Kohn, which is restricted to Asiatic and European regions (Petróczy et al., 2012; Villarino et al., 2013; Rungjindamai et al., 2014; Abate et al., 2018a) and has been reported as less aggressive, plus *Monilinia mumecola* Y. Harada, Y. Sasaki and T. Sano and *Monilinia yunnanensis* M.J. Hu and C.X. Luo ex Sand.-Den. and Crous (Cox et al., 2018). Nowadays, in Europe, *M. fructicola* has largely replaced *M. fructigena*, and it co-exists with *M. laxa* on stone fruit in several European areas (Villarino et al., 2013; Abate et al., 2018a).

Impact of brown rot is remarkable, even only considering the United States, where the stone fruit market has an annual value of approximately $4.4 billion (Cox et al., 2018). The universal annual losses from disease outbreaks have been estimated at €1.7 billion (Martini and Mari, 2014). Under favorable environmental conditions, brown rot has been associated with up to 80% of the incidence of fruit loss during postharvest (Egüen et al., 2015; Teixidó, 2017). Currently, effective brown rot control in the orchard depends on integrated strategies that are largely based on fungicide sprays and cultural practices (Martini and Mari, 2014; De Curtis et al., 2019).

The genetic mechanisms underlying diversity in *Monilinia* populations and their fitness and interactions with host plants remain to be clarified. The analysis of both small- and large-scale variations among fungal genomes provides information on stable and transient genotypic and phenotypic diversity, as well as speciation, and also provides clues to their evolutionary mechanisms (Stukenbrock, 2013). Recently, we generated high-quality draft genomes of *M. fructigena* strain Mfrg269 (Landi et al., 2018), *M. fructicola* strain Mfrc123 (De Miccolis Angelini et al., 2019) and *M. laxa* strain Mlax316 (Landi et al., 2020). These *de novo* assembled genomes have improved those already available for these three species (Naranjo-Ortíz et al., 2018; Rivera et al., 2018), while providing additional genetic resources for newly released genomes (Vilanova et al., 2021). Furthermore, they represent useful sources for investigations into the evolutionary history of the *Monilinia* genus within the *Sclerotiniaceae* family, as well as into pathogenicity mechanisms and host–pathogen interactions. Comparisons of genomes and synteny analysis are also useful for investigations into phylogenetic distances (Segata et al., 2013) and for exploring the occurrence of genome rearrangement events and the extent of genome conservation (Valero-Jiménez et al., 2020).

In fungal plant pathogens and other eukaryotes, transposable elements (TEs) are internal sources of genetic variation that have recognized roles in adaptive evolution through genome diversification (Mat Razali et al., 2019). TEs tend to harbor genes that are involved in pathogenicity and host adaptation (Santana et al., 2014), and they can evolve at higher rates than the rest of the genomic sequences, to give rise to so-called “two-speed” genomes (Dong et al., 2015). On the other hand, repeat-induced-point (RIP) mutation is a fungus-specific pathway that mutates repetitive DNA sequences during the sexual cycle, and it is mainly regarded as a host-genome defense mechanism to counteract deleterious effects of TEs (Hane et al., 2015). RIP activity contributes to gene and genome evolution because it can result in the formation of long stretches of A-T-rich regions and allows for gradual divergence of duplicated genes (Galagan and Selker, 2004). This is known for certain effector and avirulence genes that have important roles in plant–fungus interactions, to contribute to virulence and determination of host range (Plissonneau et al., 2016; Deng et al., 2017). The necrotrophic lifestyle typically involves secretion of cell-wall-degrading enzymes and toxic metabolites to destroy tissues, to degrade plant cell-wall components, and to start infection. However, recent studies have revealed more complex pathways of pathogen interactions with host plants, like for *Botrytis cinerea* Pers. and *Sclerotinia sclerotiorum* (Lib.) de Bary (e.g., van Kan et al., 2014; Kabbage et al., 2015; Rajarammohan, 2021).

The availability of fungal genomes also provides an opportunity to identify biosynthetic gene clusters (BGCs), which comprise physically clustered groups of two or more genes that encode enzymes involved in biosynthetic pathways that produce secondary metabolites. While these secondary metabolites are not directly associated with the growth of the microorganism, they can mediate a variety of microbe-host and microbe-microbe interactions by acting as bioactive compounds, metal-transporting agents and quorum-sensing molecules (Demain and Fang, 2000). Further, they are regarded as a source of natural products that have several possible uses (Cimermancic et al., 2014).

In the present study, we performed the first interspecies comparative analysis by using single representative genomes of *M. fructicola* (NCBI:txid38448) strain Mfrc123 (CBS 144850), *M. fructigena* (NCBI:txid38457) strain Mfrg269 (CBS 145095), and *M. laxa* (NCBI:txid61186) strain Mlax316 (CBS 144852). Here, we explored: (i) large-scale phylogenomic relationships between the three *Monilinia* strains and between representative species within the order *Helotiales*; (ii) syntenic relationships among the *Monilinia* genomes, to compare their structural variations; (iii) the abundance and evolutionary dynamics of their TEs; (iv) the genome-wide occurrence and extent of RIP mutations; and (v) their effector genes, CAZymes and secondary metabolite gene clusters. The well-defined genomes of *B. cinerea* and *S. sclerotiorum* as closely related species belonging to the *Sclerotiniaceae* family were used as references.

## Materials and Methods

### Phylogenetic Analysis

Single representative genome sequences of 23 species and 10 families within the order *Helotiales* were used for the phylogenetic analysis. These included the three *Monilinia* genomes reported in this study and 20 genomes retrieved in the whole-genome shotgun (WGS) database in GenBank, which were selected from among those *Helotiales* species that have high levels of completeness and annotations of the protein sequences (accessed April 12, 2019). A complete list of the genomes used is given in Supplementary Table 1. Single-copy orthologous proteins within *Sclerotiniaceae* or within *Helotiales* were identified with the software package GET_HOMOLOGUES v3.2.1 (Contreras-Moreira and Vinuesa, 2013), which clusters homologous protein families using the bidirectional best-hit, COGtriangles (-G -t 0 -D) and OrthoMCL (-M -t 0 -D) algorithms. A core-cluster was obtained by running the “compare_clusters.pl” script from the clusters generated by each of the two algorithms.

A phylogenetic tree was then constructed using the orthologous protein families identified by GET_HOMOLOGUES, which were then concatenated and aligned using MegAlign Pro (Lasergene v. 15.0.1; DNASTAR Inc., Madison, WI, United States) with the MAFFT algorithm (BLOSUM30 scoring matrix) and default settings. The tree was inferred using the maximum likelihood method implemented in MEGA7 (Kumar et al., 2016), based on the JTT matrix-based method (Jones et al., 1992) set to default parameters. Statistical support for the branches was evaluated using bootstrap analysis (Felsenstein, 1985) with 1,000 replicates.

### Synteny

Orthology-based conserved syntenic blocks among the genomes of the three *Monilinia* strains and *B. cinerea* and *S. sclerotiorum* (chosen as closely related outgroup species in the *Sclerotiniaceae* family) were analyzed using the CoGe computational pipeline^2^ (Lyons and Freeling, 2008).

The SynMap tool (Lyons et al., 2008; Castillo et al., 2018) was used to identify syntenic regions and large-scale changes in genome organization. Specifically, for each genome dot plot, comparisons were performed in pairs. Tandem gene duplicates were identified and filtered by using the blast2raw programme. Orthologous genes were identified using the BlastZ algorithm, and collinear series of syntenic genes were identified using DAGchainer (Haas et al., 2004), with the “relative gene order” option and the maximum distance between two matches parameter set to 5. Syntenic Path Assembly (SPA) (Lyons et al., 2011) in the SynMap tool was used to generate a whole genome assembly based on synteny. The rate of synonymous substitution (Ks) values was calculated as the temporal calculation of synteny for the detected syntelogous gene pairs using the Needleman–Wunsch algorithm (Needleman and Wunsch, 1970) in the CodeML software (Haug-Baltzell et al., 2015) of the PAML package (Yang, 1997), integrated in SynMap. Ks values were plotted as a histogram, and a color scheme was applied across the histogram. We further estimated the Ks values by classifying five evolutionary classes, from the younger to the older orthologous gene pairs: (1) Ks divergence ≤ 0.2; (2) 0.21 ≤ Ks ≤ 0.4; (3) 0.41 ≤ Ks ≤ 1.0; (4) 1.1 ≤ Ks ≤ 79; and (5) Ks ≥ 80. Microsynteny analysis of specific regions was performed using the SynFind (Tang et al., 2015) and GEvo (Lyons et al., 2008) tools in CoGe. In detail, we analyzed the syntenic relationships among specific genes using genes from one genome against the other analyzed genomes as the query. Then high-resolution analysis of neighboring genomic regions was performed using GEvo for comparison of multiple genomic regions. In addition, we used SynFind to estimate syntenic depth among genomes, i.e., the number of times a region is syntenic to target genome regions, and to suggest the orthologous and paralogous or co-orthologous genomic regions. SynFind analysis was performed using the BLAST-variant with a gene window size of 40 and setting 4 as the minimum number of genes.

### Transposable Elements

TEs in the three *Monilinia* genomes, as well as in the *B. cinerea* and *S. sclerotorium* genomes, were identified through both *de novo* and homology searching methods. As a first step, *de novo* repeat detection was performed on the assembled genomes using the software package RepeatModeler, version 1.0.10^3^ (Smit and Hubley, 2015), which integrates the following programmes: rmblastn v. 2.2.28; RECON v. 1.08; Tandem Repeats Finder v. 4.07b; and RepeatScout v. 1.0.5 (Benson, 1999; Bao and Eddy, 2002; Price et al., 2005; Smit et al., 2015). The sequences identified were then classified using CENSOR^4^ (Jurka et al., 1996; Kohany et al., 2006) according to the “fungi” TEs database (Kapitonov and Jurka, 2008), to obtain genome-specific libraries. For the homology-based approach by searching in TE repositories, RepeatMasker (v 4.0.7) was used to annotate the newly identified repeat elements on the genome assembly of each strain using the resulting library merged with libraries included in RepeatMasker (RepBase-20181026 library; RepeatMaskerLib.h5, Df Dfam, v 3.2; Date: July 2, 2020; Families: 318,520) to classify the unknown TE families and subfamilies, and to generate the complete annotations.

To estimate TE “age” and transposition history in the *Monilinia* species, we performed copy-divergence analysis of TEs based on their Kimura 2-parameter distances (*K*-values) (Kimura, 1980), as determined using calcDivergenceFromAlign.pl and createRepeatLandscape.pl (available on RepeatMasker util directory) on the alignment files. The proportions (%) and normalized numbers of TEs in each genome were calculated according to their classes.

With the aim to predict potentially active (autonomous) or degenerate (non-autonomous) TEs (Wicker et al., 2007; Santana et al., 2012), the presence of coding regions was assessed according to the following strategies. Overlap between TEs and annotated genes were detected using CLC Genomics Workbench and were submitted to InterProScan for conserved domain searching using the OmixBox 2.0.10 platform (Conesa et al., 2005). In addition, open reading frames (ORFs) were predicted using ORFfinder^5^ and analyzed using SmartBLAST^6^ against the UniProtKB/Swiss-prot database provided by the National Center for Biotechnology Information (NCBI^7^). The TEs selected were analyzed using the GEvo and FeatView tools on the CoGe platform to find their position features on the genomic sequences.

### Repeat-Induced Point Mutation

The whole-genome sequences of *M. fructicola*, *M. fructigena*, and *M. laxa* were subjected to repeat-induced point mutation (RIP) analysis using the RIPper programme (van Wyk et al., 2019), which measures changes in the frequencies of the targeted dinucleotides (substrates) and products that result from RIP. These analyses were performed using a 1,000-bp window size and 500-bp step size, with the default cut-off settings for the RIP parameters. Large RIP-affected regions (LRARs) were identified as genomic regions longer than 4,000 bp that included at least seven consecutive RIP-positive windows. For comparative purposes, all of these analyses were further performed on the genomes of *B. cinerea* and *S. sclerotiorum*. Changes in gene density for RIP-affected regions were investigated using the CLC Genomics Workbench. Dinucleotide frequencies were calculated using the RIPCAL tool (Hane and Oliver, 2008) with the default settings.

BLASTP searches (*E*-value < 1 × 10^–5^) were used to identify genes encoding proteins involved in the RIP pathway in each *Monilinia* genome. These included homologs of the key enzymes described in *Neurospora crassa*, 5-cytosine methyltransferase RID (RIP deficient) and DIM-2 (defective in methylation), the RIP-associated cofactors DIM-3, -5, -7, -8, and -9, and HP-1 (heterochromatic protein) (Clutterbuck, 2011). Their expression in each *Monilinia* species was evaluated by exploiting RNA-seq data (De Miccolis Angelini et al., 2018).

### Effectors, CAZymes, and Secondary Metabolite Gene Clusters

Secreted proteins in the proteome of each strain were identified by selecting sequences with a signal peptide as predicted by SignalP 5.0 (Armenteros et al., 2019), and no multiple transmembrane helices or a single helix in the N-terminal 60 residues signal peptide, as predicted by TMHMM 2.0 (Krogh et al., 2001). Selected sequences were submitted to InterProScan annotation in OmicsBox 2.0.10. Effector protein candidates for *M. fructicola*, *M. fructigena* and *M. laxa* were predicted using the EffectorP 1.0 (Sperschneider et al., 2016) and EffectorP 2.0 (Sperschneider et al., 2018) prediction tools^8^. A combined EffectorP 1.0/2.0 classifier was finally used to achieve the highest prediction accuracy, to limit false-positive results. Candidate effector proteins were screened for functional annotation based on analysis of sequence homology and searches for conserved motifs, using the amino-acid sequences as an input and an E-value cut-off of 0.001. Carbohydrate-active enzymes (CAZymes) were annotated using dbCAN2 meta server (Zhang et al., 2018). Only CAZymes that were identified by at least two of the three tools, HMMER, DIAMOND and Hotpep, were selected for further analysis. Ortholog clusters of the predicted effectors and CAZymes were identified using OrthoVenn2 (Xu et al., 2019).

Putative BGCs for each *Monilinia* strain were predicted using AntiSMASH 5.2.0^9^ (Blin et al., 2019). The web-based software SMURF^10^ (Khaldi et al., 2010) was used to detect further potential gene clusters. The default parameters were used for both of these analyses. Furthermore, RNA-seq data (De Miccolis Angelini et al., 2018) were used to identify co-regulated gene clusters using FunGeneClusterS^11^ (Vesth et al., 2016), with the parameter setting as follows: window-size = 3, gene skipping = 0 and Pearson correlation.

## Results

### Phylogenetic Analysis

**FIGURE 1:**
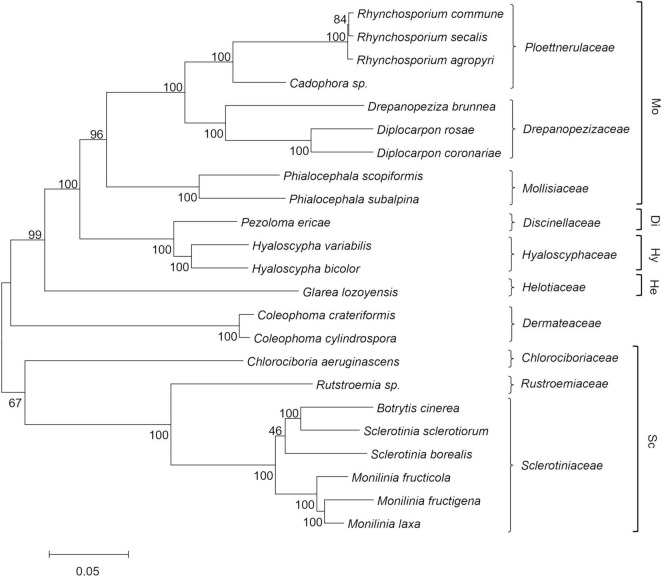
Maximum likelihood phylogenetic tree based on 129 concatenated orthologous single copy proteins recognized using GET_HOMOLOGOUS from 23 *Helotiales* genomes, including the three newly sequenced *Monilinia* genomes. Labels include the taxonomic name and the family level classification (right), according to the CABI database (http://www.speciesfungorum.org/Names/fundic.asp). Families are included within the informal “mollisioid” (Mo), “discinelloid” (Di), “hyaloscypheloid” (Hy), “helotioid” (He), and “sclerotinioid” (Sc) clades recognized by Johnston et al. (2019). The tree was inferred using the maximum likelihood method and the JTT matrix-based model. The evolutionary distances (number of amino-acid substitutions) were computed using the Poisson correction method, with elimination of all positions containing gaps and missing data. Bootstrap tests were carried out with 1,000 replicates.

The results from the phylogenetic analysis are shown in Figure 1 as a maximum likelihood phylogenetic tree that was obtained using 129 single-copy orthologous proteins across 23 *Helotiales* genomes. The taxonomic relationships inferred by this analysis are congruent with the current family level taxonomy and with the family clades within *Helotiales* defined by Johnston et al. (2019). A first group of families that included *Mollisiaceae*, *Drepanopezizaceae*, and *Ploettnerulaceae* were grouped in the “mollisioid” clade with 96% bootstrap support. The family *Discinellaceae* member of the “discinelloid” clade was closely related to *Hyaloscyphaceae* (“hyaloscyphoid” clade) and distant from *Helotiaceae* (“helotioid” clade) and *Dermateaceae*. As expected, the *Monilinia* species were closely related to *B. cinerea* and *Sclerotinia* species in the *Sclerotiniaceae* family, which together with the sister family *Rutstroemiaceae* and the more distant *Chlorociboriaceae*, are recognized as members of the “sclerotinioid” clade. A strong molecular phylogenetic structure was observed with bootstrap values >95% for most tree key branches, with a few branches showing lower bootstrap values. The relationship among *Sclerotinia borealis* and the closest *Sclerotinia* and *Botrytis* species within *Sclerotiniaceae* was poorly supported by the bootstrap analysis. The phylogenetic distance inside *Sclerotiniaceae* was also confirmed by maximum likelihood analysis based on a larger set (2,006) of single-copy ortholog proteins identified among the same species of the family, with *Glarea lozoyensis* used as an outgroup (data not shown).

### Synteny

Syntenic gene pairs among the *Monilinia* genomes were identified using SynMap. There were totals of 13,749, 12,424 and 10,800 coding genes for *M. fructicola*, *M. laxa* and *M. fructigena*, respectively. *M. fructicola* returned 9,015 to 7,765 syntenic genes when paired with *M. laxa* and *M. fructigena*, respectively, while 7,813 genes were syntenic between the *M. laxa* and *M. fructigena* genomes. The overall comparison highlighted 7,398 genes shared among these genomes (Supplementary Figure 1). The *Sclerotiniaceae* inter-genus comparison showed high syntenic levels: 7,930, 7,827 and 6,824 of the 13,703 coding genes of *B. cinerea* were syntenic with *M. laxa*, *M. fructicola* and *M. fructigena*, respectively, while 7,640, 7,572 and 6,623 of the 11,130 *S. sclerotiorum* genes were syntenic with *M. laxa*, *M. fructicola* and *M. fructigena*, respectively. Overall, 5,715 coding genes were shared by the five fungal strains tested (Supplementary Figure 1).

**FIGURE 2:**
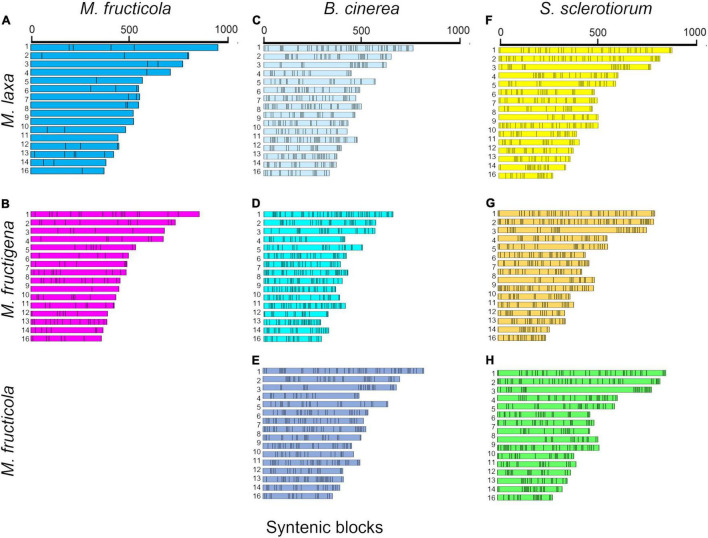
Syntenic blocks shared between *M. fructicola* and *M. laxa* **(A)** or *M. fructigena* **(B)**, *B. cinerea* and *M. laxa* **(C)**, *M. fructigena* **(D)** or *M. fructicola* **(E)**, and *S. sclerotiorum* and *M. laxa* **(F)**, *M. fructigena* **(G)** or *M. fructicola* **(H)**. Colored boxes represent syntenic blocks along the scaffolds/chromosomes. The length of each scaffold/chromosome is based on the number of genes included in the syntenic blocks, which is an approximation of the actual size. As expected, the more distinct the species, the more fragmented the syntenic blocks. Scaffolds or chromosomes not showing syntenic genes with the other species are not shown.

The investigation of collinear orthologous genes revealed a highly conserved gene order among the *Monilinia* genomes. In particular, the syntenic collinear blocks identified between *M. fructicola* and *M. laxa* were extensively conserved, with an average of 180 genes per 50 syntenic blocks. Then, 136 and 143 syntenic blocks with an average of about 50 genes per block were detected in the *M. laxa/M. fructigena* and *M. fructicola/M. fructigena* comparisons, respectively. As expected, greater fragmentation of syntenic blocks was detected by comparing the *Monilinia* genomes with *B. cinerea* and *S. sclerotiorum*. In the comparisons with *B. cinerea*, a range of 411–419 syntenic blocks were counted, while they were in the range 364–392 in comparisons with *S. sclerotiorum* (Figure 2, Table 1, and Supplementary Table 2).

**TABLE 1:** Collinear syntenic blocks obtained by SynMap analysis showing the overall synteny in the pairwise alignments among the *Monilinia fructicola*, *M. fructigena*, *M. laxa*, *B. cinerea* and *S. sclerotiorum* genomes.

Pairwise comparison	Pairs of genes involved	Syntenic blocks	Average genes per syntenic block
*M. fructicola/M. fructigena*	7,765	143	54.3
*M. fructicola/M. laxa*	9,015	50	180.3
*M. laxa/M. fructigena*	7,813	136	57.4
*B. cinerea*/*M. fructicola*	7,827	411	19.1
*B. cinerea*/*M. fructigena*	6,824	419	16.3
*B. cinerea*/*M. laxa*	7,930	414	19.2
*S. sclerotiorum*/*M. fructicola*	7,572	364	20.9
*S. sclerotiorum*/*M. fructigena*	6,623	388	17.1
*S. sclerotiorum*/*M. laxa*	7,640	368	20.8
*S. sclerotiorum*/*B. cinerea*	8,442	311	27.2

**FIGURE 3:**
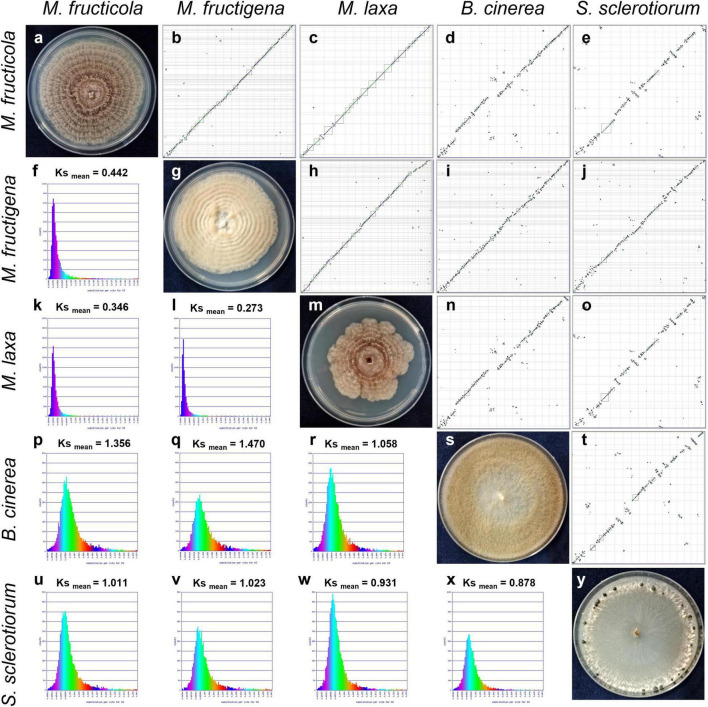
Syntenic dot plots **(b**–**e,h**–**j,n,o,t)** and synonymous (Ks) analysis **(f,k,l,p**–**r,u**–**x)** of the *M. fructicola*, *M. fructigena*, *M. laxa*, *B. cinerea* and *S. sclerotiorum* genomes. 7-day-old cultures on PDA are shown along the diagonal **(a,g,m,s,y)**. In the dot-plot graphs, horizontal or vertical gray lines delineate scaffolds ordered and oriented according to synteny, using the “Syntenic Path Assembly” option. Each dot represents an orthologous gene pair. Syntenic gene pairs are plotted with color based on the same forward order (green) or inverted matching (blue). Gray squares underline syntenic blocks. In the Ks graphs, younger syntelogs (lower numbers of synonymous changes) are purple blue (left), while older syntelogs (higher numbers of synonymous changes) are cyan-yellow-green (right).

The dot plots of whole genome synteny performed with SPA tools are shown in Figure 3, while detailed SPA mapping results are reported in Supplementary Table 2. A high syntenic level was observed among the *Monilinia* genomes, while the syntenic dot plots that included *B. cinerea* and *S. sclerotiorum* showed many discontinuities linked to highly fragmented syntenic blocks (Figure 3).

The dot plot investigation between the *M. fructicola* and *M. laxa* genomes showed discontinuities of diagonal lines in several genomic regions included in syntenic blocks (Supplementary Figure 2 and Supplementary Table 2). Microsynteny investigation showed that these genome sequences corresponded to AT rich (GC% < 20%) non-coding homologous regions that ranged from ∼70,000 to 110,000 bp. In these regions, high densities of non-autonomous/degenerated TEs were identified. Similar homologous regions were absent in *M. fructigena*. For each of these regions, the flanking genes in the *M. fructicola* and *M. laxa* genomes were syntenic with the genes located at the terminal positions of two *M. fructigena* scaffolds, which were adjacent to non-syntenic genes annotated as active TEs. These consisted of RNA-directed DNA polymerase (RdDP) linked to a non-long terminal repeat (non-LTR)/Tad1-2_ACap element, which showed the highest GC content (>62%) of the codon wobble positions, suggesting probable horizontal gene transfer, and of hypothetical proteins linked to DNA/Mariner transposons or LTR/retrotransposons (Supplementary Figure 2 and Supplementary Table 2).

**FIGURE 4:**
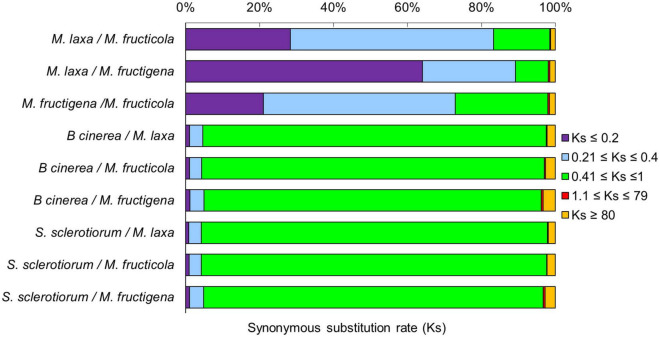
Frequency of synonymous Ks substitutions according to the *M. fructicola, M. fructigena, M. laxa*, *B. cinerea* and *S. sclerotiorum* genome relationships. Purple (Ks ≤ 0.2) is indicative of younger mutations, cyan (0.21 ≤ Ks ≤ 0.4) of much longer divergence, green (0.41 ≤ Ks ≤ 1) of older mutations, red (1.1 ≤ Ks ≤ 79) of ancient whole-genome duplication events, while orange (Ks ≥ 80) is interpreted as noise.

The rates of coding sequence evolution in syntenic regions were measured by synonymous changes (Ks) (Figure 3). Overall, one distinct peak was detected on the Ks histogram plots, which represent orthologous syntenic regions that follow taxa divergence during speciation. The Ks peak of *Monilinia* intra-genus comparisons was characterized by the prevalence of the blue-purple color, which suggested that there were no considerable changes in mutation rates at the genome-wide level. The mean Ks value between *M. fructigena/M. laxa* (Ks_mean_ = 0.273) was lower than both of those for *M. laxa/M. fructicola* (Ks_mean_ = 0.346) and *M. fructigena/M. fructicola* (Ks_mean_ = 0.442). Although the coding gene sequences between the three *Monilinia* genomes were very well conserved, these data support the hypothesis that *M. fructigena* is closer to *M. laxa* than to *M. fructicola*. The inter-genus comparison showed a prevalence of the green-cyan color, which emphasizes higher numbers of synonymous changes than older evolutionary changes. In particular, the highest Ks values in pairwise comparisons of the *Monilinia* genomes with *B. cinerea* and *S. sclerotiorum* were detected for *M. fructigena* (Ks_mean_ = 1.470, 1.023, respectively) and *M. fructicola* (Ks_mean_ = 1.356, 1.011, respectively), while the lowest were for *M. laxa* (Ks_mean_ = 1.058, 0.931, respectively) (Figure 3). These results suggest that the *M. laxa* genome is genetically closer to those of *B. cinerea* and *S. sclerotiorum*, as compared to the *M. fructigena* and *M. fructicola* genomes. An analysis of gene distribution in classes of Ks values (Figure 4) showed that homologous gene pairs with Ks ≤ 0.2 were more than 60% in the *M. laxa/M. fructigena* comparison and were as low as 28.3% and 21.0% in comparisons between *M. fructicola* and *M. laxa* or *M. fructigena*, respectively. This distribution of Ks values suggested a more ancient separation of *M. fructicola* from the other two *Monilinia* species. The frequency of homologous gene pairs with Ks ≤ 0.2 was lower than 1% in comparisons between each *Monilinia* species and *B. cinerea* or *S. sclerotiorum*.

The highest proportions of genes with a syntenic depth of 1 (indicative of a single orthologous genomic region) ranged from 80 to 95% in all of the pairwise comparisons among the tested *Monilinia* genomes, and from 57 to 73% in the comparisons with *B. cinerea* and *S. sclerotiorum*, respectively. A syntenic depth > 1 was more frequently detected (up to 17–18%) in the pairwise comparisons with *M. fructigena* used as the reference genome (Table 2).

**TABLE 2:** Syntenic depth table for all pairwise comparisons between query and target genomes of *M. fructicola*, *M. fructigena*, *M. laxa*, *B. cinerea* and *S. sclerotiorum* according to SynFind analysis.

Pairwise comparison	Syntenic depth				
(query/target)	0	1	2	3	4
*M. fructicola/M. fructigena*	0.15	80.99	17.74	1.12	0.01
*M. fructicola/M. laxa*	0.15	93.97	5.77	0.12	0.00
*M. laxa/M. fructicola*	0.15	95.69	4.16	0.00	0.00
*M. fructigena/M. fructicola*	1.27	90.12	8.43	0.18	0.00
*M. laxa/M. fructigena*	0.15	80.29	18.38	1.17	0.00
*M. fructigena/M. laxa*	1.26	91.09	7.59	0.06	0.00
*M. fructicola/B. cinerea*	0.21	66.77	30.32	2.48	0.17
*B. cinerea/M. fructicola*	0.63	67.03	28.15	3.98	0.21
*M. fructigena/B. cinerea*	1.31	63.21	29.91	4.99	0.57
*B. cinerea/M. fructigena*	1.17	63.39	31.77	3.52	0.15
*M. laxa/B. cinerea*	0.15	64.83	30.81	3.88	0.27
*B. cinerea/M. laxa*	0.77	67.69	27.32	3.92	0.31
*M. fructicola/S. sclerotiorum*	0.16	73.45	24.02	2.06	0.30
*S. sclerotiorum/M. fructicola*	0.33	63.94	31.33	4.30	0.10
*M. fructigena/S. sclerotiorum*	1.39	67.39	27.28	3.84	0.10
*S. sclerotiorum/M. fructigena*	0.71	57.08	36.26	5.78	0.17
*M. laxa/S. sclerotiorum*	0.15	69.64	27.53	2.48	0.20
*S. sclerotiorum/M. laxa*	0.35	62.85	32.43	4.26	0.11
*B. cinerea/S. sclerotiorum*	0.59	75.44	21.21	2.61	0.14
*S. sclerotiorum/B. cinerea*	0.33	68.07	27.08	4.21	0.32

### Transposable Elements

**FIGURE 5:**
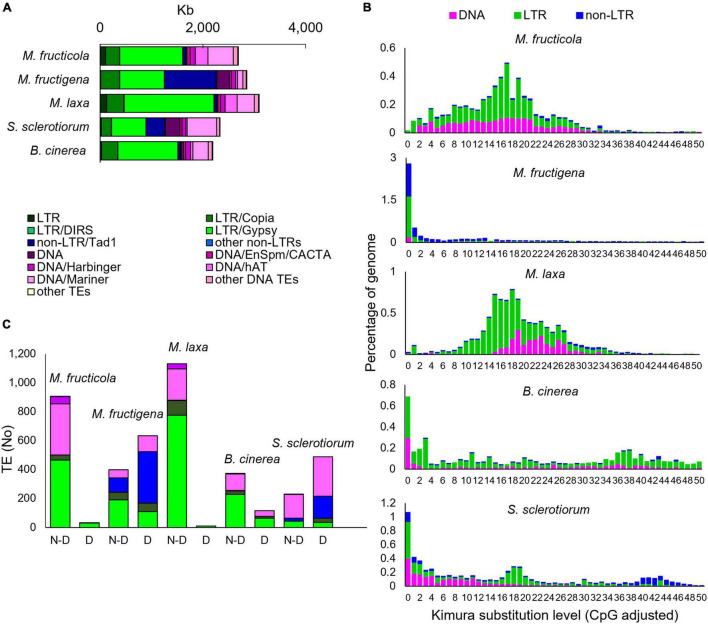
Transposable elements (TEs) in the *M. fructicola*, *M. fructigena, M. laxa*, *B. cinerea*, and *S. sclerotiorum* genomes. **(A)** Major groups of TEs identified. **(B)** Comparisons of TE landscapes. Bar charts: ordinate illustrates proportion of genome occupied by each TE, and abscissa illustrates genetic divergence from the consensus (Kimura substitution level) observed among copies of each TE. Copies clustering on the left of the graph do not diverge very much from the consensus sequence of the element and potentially correspond to recent copies, while sequences on the right might correspond to ancient, degenerate copies. **(C)** Analysis of autonomous and non-autonomous TEs. Total TEs with length >500 bp without (N-D) or with (D) conserved functional domains are shown. The Repbase classification system was used (Bao et al., 2015).

For the *Monilinia* genome sequences, approximately 2.85 Mb (6.6%) of the 43.13 Mb of *M. fructigena*, 2.70 Mb (6.1%) of the 44.05 Mb of *M. fructicola* and 3.10 Mb (7.2%) of the 42.81 Mb of *M. laxa* consisted of TEs. Lower frequencies were detected in *B. cinerea* (2.19 Mb, 5.1% of 42.81 Mb) and *S. sclerotiorum* (2.3 Mb, 6.0% of 38.90 Mb) (Figure 5A and Supplementary Table 3). A strong heterogeneity was detected in the TE typology. Some TEs (e.g., LTR/Gypsy-2_PaPe-I or DNA/Mariner1_AO) occurred in large quantities and sizes in *M. fructicola* and *M. laxa*, but were absent or present as low numbers of small fragments (<500 bp) in *M. fructigena*, *B. cinerea* and *S. sclerotiorum* (Supplementary Table 4).

The Class I TEs was the most represented for all the species tested. In the *Monilinia* strains, the frequency ranged from 3.8% in *M. fructicola* to 5.3% in *M. laxa* and *M. fructigena*, and it was 3.7% and 3.3% of the genomes of *B. cinerea* and *S. sclerotiorum*, respectively. Six superfamilies of non-LTR retrotransposons were identified. The highest percentage was detected in the *M. fructigena* genome, due to the abundance of non-LTR/Tad1 (2.3%) compared to the other genomes (0.1–0.9%). LTR retrotransposons were mainly represented by the ubiquitous LTR/Gypsy superfamily followed by the LTR/Copia superfamily, and were predominant in *M. laxa*, *M. fructicola*, *B. cinerea* and *S. sclerotiorum*. LTR/Gypsy occupied 4.1% of the *M. laxa* genome and from 1.7% to 2.8% of the others. Twenty superfamilies of Class II DNA TEs were detected in all of the analyzed genomes, with very few exceptions. Overall, the *M. fructicola* genome showed the highest proportion of DNA TEs (2.4%), like *S. sclerotiorum* (2.7%). DNA/Mariner and DNA/hAT were the most represented superfamilies in *M. fructicola*, *M. laxa*, *B. cinerea* and *S. sclerotiorum*, while lower frequencies of these elements were detected in the *M. fructigena* genome (Figure 5A and Supplementary Table 3).

*Monilinia fructicola* showed the highest frequencies (TE counts/Mb genome) of Class I retrotransposons and Class II DNA TEs in the genome, although the total fraction (Mb) of the genome occupied by TEs was lower than in the other strains. This suggests a greater fragmentation of the annotated TEs in this genome.

To investigate TE dynamics, the distribution of TEs in the genome of each species were assessed based on their *K*-values (Figure 5B). The TE landscape in *M. fructigena* showed a burst of TE insertions where the highest accumulation occurred more recently (90% of TEs with *K*-values < 4) compared with *M. laxa* and *M. fructicola*, which showed one major peak corresponding to *K*-values of 15–20. In *M. laxa*, after the main burst, all of the transposon categories were contracted to lower levels, whereas the Class II DNA TEs that were more consistently detected in *M. fructicola*. *B. cinerea* and *S. sclerotiorum* genomes showed a recent TE burst that was similar, although to a lesser extent, to that in *M. fructigena*. The two genomes showed both an older burst (*K*-value = 34–42) mostly involving LTR and DNA TEs in *B. cinerea* and non-LTR elements in *S. sclerotiorum*. Moreover, a younger burst of TE insertion events (*K*-value = 18–20) that mainly involving LTR elements was observed in *S. sclerotiorum*.

The prediction of potentially active or degenerated TEs revealed that non-autonomous TEs and TE remnants (<200 bp in size) massively overlapped with genomic regions annotated as coding genes. TEs < 500 bp, however, had no detectable TE-related domains, whereas differences in the levels of TEs ≥ 500 bp with or without functional conserved domains were observed among the genomes analyzed (Figure 5C). The complete list of the TE types, the relative functional domains and the annotated genes is given in Supplementary Table 4. Active TEs with ORFs coding for proteins required for transposition were rarely detected in *M. laxa* and *M. fructicola*, while they were more frequent in the other genomes, with the highest proportion in *M. fructigena*, followed by *S. sclerotiorum* and *B. cinerea*. None of the 938 and 1,141 TEs ≥ 500 bp (up to ∼10,000 bp in size) that were identified in *M. fructicola* and *M. laxa*, respectively, overlapped with annotated gene sequences. Only 9 TEs in *M. fructicola* and 39 in *M. laxa* contained TE-related domains that mainly belonged to LTR/Gypsy. On the contrary, 641 of 1,031 TEs ≥ 500 bp identified in *M. fructigena* overlapped with 739 genes, with 86% of these showing more than 50% overlap. Among 633 TEs with at least one TE-related domain identified in *M. fructigena*, 562 were in-gene TEs and 96 were not in-gene TEs. In a similar way, 290 of 719 TEs ≥ 500 bp in *S. sclerotiorum* overlapped with 325 genes, with 73% of them showing more than 50% overlap; among 483 TEs carrying at least one TE-related domain, 284 were in-gene TEs and 199 were not in-gene TEs. Twenty-one of 604 TEs ≥ 500 bp in *B. cinerea* overlapped with 22 genes, with the 80% of them showing more than 50% overlap; among 110 carrying at least one TE-related domain, 9 were in-gene TEs and 106 were not in-gene TEs. In the *M. fructigena*, *S. sclerotiorum*, and *B. cinerea* genomes, LTR/Gypsy elements overlapped with genes crucial for retrotransposition; i.e., structural (Gag), structural-catalytic polyprotein (Gag-Pol), derivate reverse transcriptase, integrase and retrotransposon nucleocapsid. The LTR/Copia overlapped frequently with genes coding for ribonuclease H, retrotransposon hobase, Gag, Gag-Pol, reverse transcriptase and integrase. The non-LTR/Tad1 in the *M. fructigena* genome largely overlapped genes coding for RNA-directed DNA polymerase from transposon X-element, while in *S. sclerotiorum* and *B. cinerea*, they mainly overlapped with hypothetical proteins.

### Repeat-Induced Point Mutation

**FIGURE 6:**
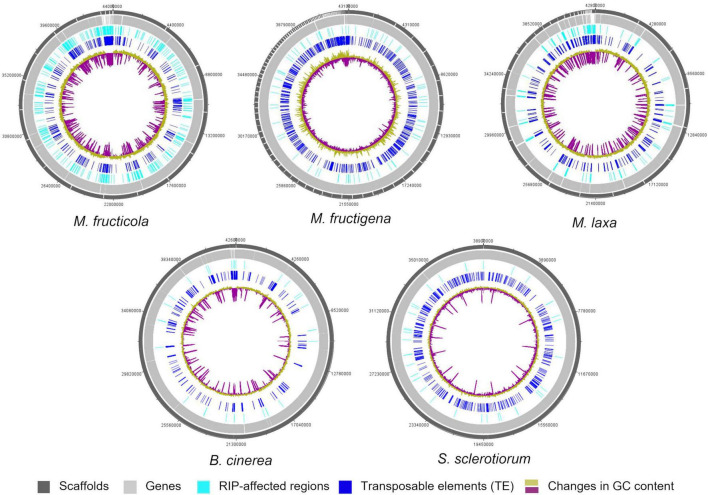
Circular representation of genetic features of the *M. fructicola*, *M. fructigena, M. laxa, B. cinerea*, and *S. sclerotiorum* genomes. Outer circles: distribution of scaffolds, annotated genes, regions affected by Repeat-induced point mutation (RIP) and transposable elements (>500 bp) across the length of the genome assembly in the five species. The color key is shown at the bottom of the map. Inner circle: changes in GC content calculated using a 10,000-bp window and 200-bp increments.

Only small proportions of the three *Monilinia* genomes were RIP positive, with RIP product indices > 1.1, RIP substrate indices < 0.75 and RIP composite index ≥ 0. The extent to which RIP mutations were recorded varied slightly among the genomes, with ranking as follows: *M. fructicola* > *M. laxa* > *M. fructigena*. These were, in any case, higher compared to *B. cinerea* and *S. sclerotiorum* (Table 3). In *M. fructicola*, 2.3% of the entire genome assembly contained RIP mutations with proportions varying from 1.5% to 3.4% in 16 large scaffolds (VICG01000001-VICG01000016), which appear to represent the core chromosomes, and from 9.3% to 10.4% in the remaining small scaffolds (VICG01000017-VICG01000019) (Supplementary Table 5). RIP activity was also recorded in the scaffold VICG01000020, which is made up of repeat units of ribosomal DNA, and where it was limited to intergenic spacers. Fewer windows suggestive of RIP (1.0%) were detected in the *M. laxa* genome, which ranged from 0.2% to 1.3% in large scaffolds (VIGI01000001-VIGI01000017). Even fewer RIP regions were recorded in the *M. fructigena* genome, with a small number of affected windows that ranged from 0.0% to 0.6% in most of the assembled scaffolds, corresponding to 0.4% of the whole genome sequences. In the *B. cinerea* and *S. sclerotiorum* genomes, RIP-affected windows were ≤ 0.1% in the core chromosomes, and 0.5% to 0.6% in the two *B. cinerea* mini-chromosomes. RIP mutations were more obviously co-localized with TE-rich regions in *M. fructicola* and *M. laxa* than in *M. fructigena* (Figure 6). Reduced GC content (17.1% in *M. fructicola*, 18.8% in *M. laxa*, 31.9% in *M. fructigena*) was recorded in genomic regions suggestive of RIP, compared to that of the entire genome (40–42%). Gene density was 2.3 and 1.9 genes per 100 kb in RIP-affected windows of *M. fructicola* and *M. laxa*, while the average contents of genes in the genomes were 27.5 and 26.1 genes per 100 kb, respectively (Table 3). Genes in these regions (121 in *M. fructicola*, 29 in *M. laxa*) were largely without functional annotations (43–55%). In *M. fructigena*, the genomic gene density (24.4 genes per 100 kb, on average) increased up to 41.8 in RIP-affected regions (Table 3). The genes contained here (*n* = 204) were mostly involved in biological processes (e.g., endonucleolytic RNA phosphodiester bond hydrolysis [GO:0090502] and DNA integration [GO:0015074]) and molecular functions (e.g., nucleic acid binding [GO:0003676] and RNA-DNA hybrid ribonuclease activity [GO:0004523]), with prevalent nuclear localization (GO:0005634) (data not shown).

**TABLE 3:** Genome-wide repeat-induced point mutation (RIP) statistics for *M. fructicola*, *M. fructigena*, *M. laxa*, *B. cinerea* and *S. sclerotiorum*.

RIP statistic	*M. fructicola*	*M. fructigena*	*M. laxa*	*B. cinerea*	*S. sclerotiorum*
Number of RIP positive windows	2,030	318	865	40	38
RIP-affected genomic proportion^1^ (%)	2.30	0.37	1.01	0.05	0.05
Average RIP composite index in RIP-affected regions	0.61	0.77	0.53	0.99	0.81
GC content of genome assembly (%)	40.8	42.1	39.8	42.0	41.6
GC content of RIP-affected regions (%)	17.1	31.9	18.8	28.4	35.7
Gene density^2^ in genome assembly	27.5	24.4	26.1	27.5	29.2
Gene density^2^ in RIP-affected regions	2.3	41.8	1.9	6.8	30.7
Number of LRARs^3^	9	0	2	0	0
Average size of LRARs (bp)	45,700	-	31,500	-	-
Range of RIP-positive windows in individual large scaffolds^4^ (%)	1.52–3.38	0.08–0.57	0.21–1.26	0.00–0.09	0.02–0.12

*^1^Proportion of genome that is RIP-affected estimated by the RIPper software using the number of windows with RIP-positive index values (product index ≥ 1.1; substrate index ≤ 0.75; composite index > 0) against the total number of investigated windows in the entire genome.*

*^2^Gene density calculated as genes per 100 kb.*

*^3^LRAR, large RIP affected regions, i.e., ≥ 4 kb consecutively affected by RIP.*

*^4^The first 16–17 larger scaffolds (>0.8 Mb in length) were considered.*

Few LRARs were identified in the genomes of *M. fructicola* (*n* = 9) and *M. laxa* (*n* = 2), with none identified in the *M. fructigena*, *B. cinerea*, and *S. sclerotiorum* genomes. The LRARs presented low GC contents and did not contain genes, with very few exceptions. For six of the LRARs predicted in *M. fructicola* and one of those predicted in *M. laxa*, fine-scale analysis of their flanking sequences revealed that they were included in large RIP-affected regions (14,000–103,000 bp in size) with marked variation in RIP index values. All of these regions were rich in TEs (mainly DNA/Mariner, DNA/hAT, LTR/Gipsy), and represented synteny breakpoint regions (Supplementary Figure 3).

Di-nucleotide frequency analysis of the RIP-affected regions identified in the *Monilinia* genomes showed the expected pattern of classical CpA→TpA type RIP mutations and the occurrence of CpG→TpA and CpC→TpT double mutations. Moreover, there was depletion of GpC, and to a lesser extent of TpC, that resulted from changes in the second nucleotides, as already reported for *B. cinerea* and *S. sclerotiorum* (Clutterbuck, 2011) (Supplementary Figure 4).

The *Monilinia* genomes contained genes that encoded homologs (with conserved domains) of the RID and DIM-2 enzymes, which are essential for RIP and RIP-associated methylation in *N. crassa*, and the associated co-factors HP-1 and DIM-3, -5, -8, -9, but not DIM-7 (Table 4). RNA-seq data provided evidence of transcription for all of these genes detected (data not shown).

**TABLE 4:** Genes putatively involved in repeat-induced point (RIP) mutations in the *M. fructicola*, *M. fructigena*, *M. laxa* genomes (vs. *N. crassa*).

Gene	Accession	Description	*M. fructicola*			*M. fructigena*			*M. laxa*		
product^1^	number		Gene identifier	*E*-value	Similarity (%)^2^	Gene identifier	*E*-value	Similarity (%)^2^	Gene identifier	*E*-value	Similarity (%)^2^
RID	XP_011392925.1	RIP defective (cytosine-C5 specific DNA methylases)	EYC84_001176	4e-62	41.1	DID88_005494	3e-59	40.4	EYC80_007134	4e-62	41.2
DIM-2	XP_959891.1	DNA methyltransferase	EYC84_008717	0.0	44.3	DID88_009984	7e-124	45.0	EYC80_007749	1e-179	35.9
DIM-3	XP_960652.1	Karyopherin (importin) alpha subunit	EYC84_007939	0.00	87.7	DID88_006995	0.00	76.0	EYC80_002110	0.00	87.7
DIM-5	XP_957479.2	Histone-lysine N-methyltransferase	EYC84_010305	2e-117	50.2	Unannotated gene on QKRW01000011.1 (612,808 to 614,129)			EYC80_009896	2e-117	50.2
DIM-7	XP_961308.2	Defective in DNA methylation-7 protein	Not detected			Not detected			Not detected		
DIM-8	XP_962347.1	DNA damage-binding protein 1	EYC84_000468	0.00	45.8	DID88_006444	0.00	44.1	EYC80_001863	0.00	47.4
DIM-9	XP_956278.3	Defective in DNA methylation-9 protein	EYC84_005276/7/8	7e-39	57.6	DID88_002795	7e-61	37.1	EYC80_005058	2e-60	36.5
HP-1	XP_957632.1	Heterochromatin protein 1	EYC84_009794	7e-60	45.6	DID88_008074	6e-31	37.2	EYC80_009694	3e-51	46.6

*^1^gene product in N. crassa.*

*^2^similarity to the N. crassa homolog.*

### Effectors, CAZymes, and Biosynthetic Gene Clusters

In *M. fructicola*, there were 759 secreted proteins, with 753 in *M. laxa* and 532 in *M. fructigena*, corresponding to 5% to 6% of the total annotated proteins. These data agree with those reported for *S. sclerotiorum* (Guyon et al., 2014), *B. cinerea* (Amselem et al., 2011; van Kan, 2017) and other *Botrytis* species (Valero-Jiménez et al., 2019). The prediction results of EffectorP 1.0 and EffectorP 2.0 and the number of proteins of each *Monilinia* strain labeled as effectors by both prediction tools are given in Table 5. The final numbers of the predicted effectors ranged from 56 (*M. fructigena*) to 68 (*M. fructicola*), which represented ∼0.5% of the total proteome of each strain, and from 8.1% (*M. laxa*) to 10.5% (*M. fructicola*) of the secretome, in agreement with the findings of Marcet-Houben et al. (2021). Orthologous clusters were identified among putative effectors identified in each *Monilinia* genome (Supplementary Figure 5). A set of 15 effector candidates had orthologs in all three genomes, while 12 were common to *M. fructicola* and *M. laxa*, 9 were common to *M. laxa* and *M. fructigena*, and 5 were common to *M. fructicola* and *M. fructigena*. Several effectors of *M. fructicola* (*n* = 36), *M. laxa* (*n* = 25), and *M. fructigena* (*n* = 27) did not have orthologs among the candidate effectors predicted in the other genomes. A list of all of the orthologous clusters and their conserved functional domains is given in Supplementary Table 6.

**TABLE 5:** Number of candidate effectors in the *Monilinia* genomes, as predicted by EffectorP (versions 1.0 and 2.0).

Species	Total proteins	Secreted proteins	Candidate effectors [n (%)]		
	(n)	(n)	EffectorP1	EffectorP2	EffectorP1 and EffectorP2
*M. fructicola*	13,749	759	94 (0.68)	97 (0.70)	68 (0.49)
*M. fructigena*	10,800	532	79 (0.73)	82 (0.76)	56 (0.52)
*M. laxa*	12,424	753	85 (0.68)	96 (0.77)	61 (0.49)

Among nine candidate effectors common to the five genomes, one was a Nep1-like protein (NLP) with a necrosis-inducing protein NPP1 domain (PF05630). The other ones comprised, for instance: a protein carrying a peptidase inhibitor I9 and peptidase S8 domains (G3DSA:3.30.70.80); a Rare lipoprotein A (RlpA) with a conserved region with a double-psi β-barrel (DPBB) fold (PF03330); a protein with a CVNH (cyanovirin-N homology) domain (PF08881); and a member of the PTHR40845 panther superfamily associated to putative effectors of fungal plant pathogens.

A pectate lyase (PF03211) was identified in the three *Monilinia* genomes and in *B. cinerea*, while a hydrophobic surface binding protein A (HsbA; PF12296) had orthologs in the three *Monilinia* genomes and in *S. sclerotiorum*. A family 2 peroxidase (PF01328) and a FK506-binding protein (FKBP; PF00254) had orthologs in all three of the *Monilinia* genomes. The remaining orthologs identified in more than one genome included a protein containing a MD-2-related lipid-recognition (ML) domain (PF02221), which has been implicated in the recognition of pathogen-related products like lipopolysaccharides, a putative Egh16-like virulence factor (PF11327), a protein with a cerato-platanin domain (PF07249), and a fungal hydrophobin (PF06766).

Among candidate effectors predicted only in the *Monilinia* genomes, one contained a phosphatidylethanolamine-binding protein (PEBP; PF01161), and one carried the tuberculosis necrotizing toxin (TNT) domain from *Mycobacterium tuberculosis* (PF14021), which induces necrosis of infected cells. Effectors predicted in *M. fructicola* but not in *M. laxa* and *M. fructigena* included, e.g., a FAS1 (fasciclin-like) domain protein (SSF82153) with orthologs in *B. cinerea* and *S. sclerotinia*; a cerato-platanin-like protein (PF07249); and a protein with chitin-binding, type 1 (PF00187) and fungal cellulose binding (PF00734) domains. A candidate effector identified only in *M. laxa* and *M. fructigena* was associated to the heat-labile enterotoxin family (PF01375), while an Egh16-like virulence factor (PF11327) was predicted only in *M. fructigena*, as well as a putative GPI-anchored protein (PTHR39599), a [PSI+] induction protein 2 (PTHR40018), and cupredoxin (SSF49503, G3DSA:2.60.40.420).

CAZyme prediction for the genomes revealed 183 enzymes in *M. fructicola*, 200 in *M. laxa*, 134 in *M. fructigena*, 271 in *B. cinerea* and 224 in *S. sclerotinia*, corresponding to 24.1%, 26.6%, 25.2%, 25.0%, and 29.5% of secreted proteins, respectively. These data agree with previous reports (e.g., Amselem et al., 2011; Allan et al., 2019; Marcet-Houben et al., 2021; Vilanova et al., 2021). Overall, 87 CAZyme families were identified in the predicted secretomes, including 51 glycoside hydrolases (GHs), 11 auxiliary activities (AAs), 11 carbohydrate esterases (CEs), 8 glycosyltransferases (GTs), and 3 polysaccharide lyases (PLs), as well as 9 carbohydrate-binding modules (CBMs), which can potentiate CAZyme activities (Supplementary Table 7). In all of the genomes analyzed, the largest numbers of predicted CAZymes were in the GH28 family of polygalacturonases, followed by AA3 in the *Monilinia* genomes, and by GH16 in *B. cinerea* and *S. sclerotiorum*.

**FIGURE 7:**
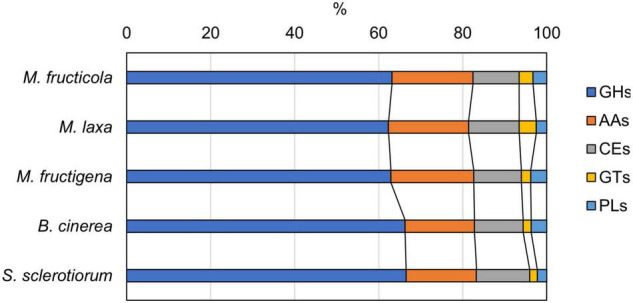
Carbohydrate-active enzyme (CAZyme) distribution predicted from the *M. fructicola*, *M. fructigena*, *M. laxa*, *B. cinerea* and *S. sclerotiorum* genomes. GHs, glycoside hydrolases; AAs, auxiliary activities; CEs, carbohydrate esterases; GTs, glycosyltransferases; PLs, polysaccharide lyases. Non-catalytic Carbohydrate-Binding Modules (CBMs) associated to CAZymes are detailed in Supplementary Table 7.

The distributions of the CAZyme classes were similar in the five genomes, with very little difference for GHs, AAs and GTs (Figure 7). CAZymes were grouped into ortholog clusters (Supplementary Figure 6 and Supplementary Table 7). A chitinase of family GH18 and two glycosyltransferases (GT15, GT31) detected in the *Monilinia* genomes did not have orthologs in either *B. cinerea* or *S. sclerotiorum*. Members of the GH28 and GT71 families and a β-(1,3)-glucanosyltransglycosylase of the GH72 family were predicted only in *M. laxa*. CAZymes unique for *M. laxa* and *M. fructicola* included, for instance: an α-1,4-galactosaminogalactan hydrolase (GH135) homologous to cell surface spherulin 4-like protein; a glycosyl hydrolase family 43, involved in degradation of plant cell-wall arabinans; and choline dehydrogenase or related flavoprotein/GMC oxidoreductase of the subfamily AA3-2, involved in lignocellulose degradation. Two AA3-2 CAZymes were common to *M. laxa*, *M. fructicola* and *B. cinerea*, with no orthologs in *M. fructigena* and *S. sclerotiorum*, as also for mannosyltransferase (GT32), α-mannosidase (GH47), β-glucosidase (GH3), and a carbohydrate esterase family 1 (CE1). An AA3-2 member and a polygalacturonase protein (GH28) were also common to *M. laxa*, *M. fructicola* and *S. sclerotiorum*, but not to *M. fructigena* and *B. cinerea*. CAZymes common to *M. laxa* and *B. cinerea* but not to *M. fructigena* included, for instance, a cell-wall glycosyl hydrolase protein (GH105), while those common to *M. laxa*, *B. cinerea* and *S. sclerotinia* included several GHs, a mannosyltransferase, two laccases of the AA1-3 subfamily and an alginate lyase protein (PL7). CAZymes common to *M. fructicola*, *B. cinerea* and/or *S. sclerotiorum* with no orthologs in *M. fructigena* and *M. laxa* included endo-1,4-β-mannosidase (GH5), α-1,3-glucanase (GH71), a member of the GH13 family, a mannosyltransferase (GT32), and a gdsl lipase acylhydrolase family protein (CE16). A mannosidase of the GH92 family (FCW CAZy) and AA enzymes were uniquely found in *M. fructigena*, while a fungal cellulose binding domain (CE15 + CBM1) was unique for *M. fructigena* and *M. laxa*.

A total of 18 putative BGCs (4 NRPS, 3 NRPS-like, 10 T1PKS, 1 Terpene) was found in the *Monilinia* genomes (Supplementary Table 8): 17 BGCs on 10 scaffolds of the *M. fructicola* genome or on 12 scaffolds of the *M. laxa* genome, and 12 BGCs on 11 scaffolds of the *M. fructigena* genome. Generally, only one BGC per scaffold was detected, although up to 4 BGCs were on the *M. fructicola* scaffold VICG01000003.1 and on the *M. laxa* scaffold VIGI01000002.1.

The cluster coded as BGC-1 contained a NRPS Sid2-like gene, putatively involved in siderophore production because of its similarity to the ferrichrome biosynthetic gene cluster of *Aspergillus oryzae* (64–75% amino acid sequence identity). The core gene of BGC-2 showed 32 to 35% identity to the NRPS of the *Aspergillus nidulans* BGC that produces the bioactive lipopeptides aspercryptins. BGC-1 and BGC-2 were found in the three *Monilinia* genomes, and the genes included showed co-regulation in *M. fructigena*. BGC-3 was common to *M. fructicola* and *M. laxa*, but not to *M. fructigena*, and contained a core gene homologous (39% identity) to the NRPS involved in biosynthesis of destruxin A (a bioactive cyclohexadepsipeptide) in *Metarhizium robertsii*. Two NRPS-like clusters common to the three *Monilinia* genomes showed homology to BGCs involved in the biosynthesis of mycotoxins, including aspirochlorine in *A. oryzae* (BGC-4; 42–44% identity) and fusaric acid in *Fusarium verticillioides* (BGC-5; 34% identity). BGC-4 included 13 to 15 co-regulated genes in each of the three *Monilinia* genomes. Two further NRPS-like BGCs that were common to the three *Monilinia* genomes were putatively involved in the biosynthesis of antibiotic compounds, as they had similarity to the myxalamid BGC from *Stigmatella aurantica* (BGC-6; 29–36% identity) and the fusarielin H from *Fusarium graminearum* (BGC-7; 25–32% identity).

**FIGURE 8:**
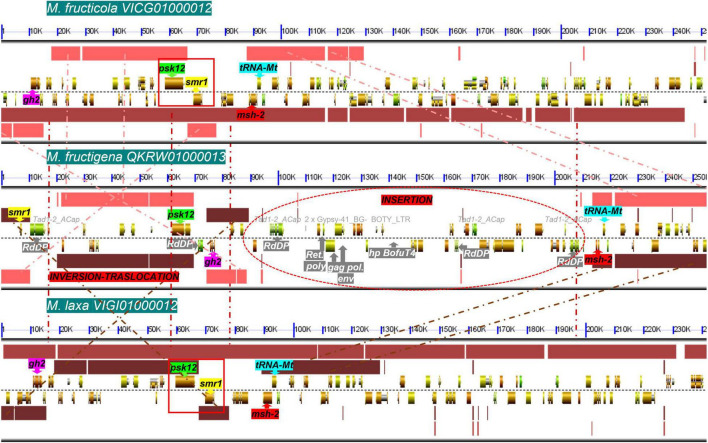
Microsynteny investigation of the genomic regions harboring the *smr1* (yellow) and *pks12* (green) genes and their flanking regions harboring the *gh2* (purple), *msh-2* (red) and *tRNA-Mt* (cyan) genes, in *M. fructicola* (scaffold VICG01000012), *M. fructigena* (scaffold QKRW0100013.1) and *M. laxa* (scaffold VIGI01000012). Syntenic blocks are represented as pink (*M. fructicola/M. fructigena*), light brown (*M. fructicola/M. laxa*) and dark brown (*M. fructigena/M. laxa*) boxes, and the homologous regions are connected by dashed lines. Like in *B. cinerea* and other *Leotiomycetes* studied, the *smr1* and *pks12* genes are clustered in a conserved module of physically linked genes (red boxes) in the *M. fructicola* and *M. laxa* genomes. In the *M. fructigena* genome, recombination events occurred that separated the *smr1* and *pks12* genes, close to genes associated to TEs (white writing in gray box) identified as new genomic insertions in non-syntenic regions. *gh2*, glycoside hydrolase family 2; *tRNA-Mt*, tRNA m(1)G methyltransferase domain; *msh-2*, DNA mismatch repair msh-2; *psk12*, polyketide synthase 12; *smr1*, sclerotial melanin regulator 1; *RdDP*, RNA-directed DNA polymerase; *gag pol env*, gag polymerase envelope; *Ret poly*, retrovirus poly; *hp*, hypothetical protein.

Among T1PKS-type BGCs, two clusters were putatively involved in DHN-melanin biosynthesis: BGC-8, containing a homolog to BcPKS12 of *B. cinerea* (88% identity), and BGC-9, containing a homolog to BcPKS13 of *B. cinerea* (87–88% identity). In *B. cinerea*, similar to other *Leotiomycetes*, the gene encoding PKS12 was physically linked with a gene encoding the transcription factor sclerotial melanin regulator 1 (SMR1) (Schumacher, 2016). In the present analysis, this *pks12*-*smr1* module appeared to be conserved within a cluster of co-regulated genes in *M. fructicola* and *M. laxa*, whereas in *M. fructigena*, the PKS12-homolog was not detected in a cluster due to a genome rearrangement that occurred in the region surrounding the *pks12* gene that resulted in a loss of synteny with *M. fructicola* and *M. laxa* (Figure 8). In more detail, the *smr1* homolog in the *M. fructigena* genome was found about 60 kb upstream from *pks12*, probably due to inversion/translocation events and insertions of active TEs (i.e., Tad1-2_ACap, Gypsy-41_BG; Boty_LTR) in the genomic region in between *smr1* and *pks12* and in the downstream region.

Three T1PKS-like BGCs were identified in all of the three *Monilinia* genomes, homologous to BGCs involved in the biosynthesis of toxicants, like the xanthone dimer with antibacterial activity neosartorin in *Aspergillus fumigatus* (BGC-10; 67% identity), the insecticidal furanocoumarin neosporin A in *N. crassa* (BGC-11; 41–47% identity), and the maleidride with herbicidal activity cornexitin (BGC-12; 34–42% identity). BGC-10 clustered genes shown to be co-regulated in *M. fructicola*, and BGC-11 and BGC-12 clustered genes shown to be co-regulated in *M. laxa*. BGC-13 and BGC-14, which are co-regulated in *M. fructicola* and *M. laxa* and absent in *M. fructigena*, were putatively involved in the biosynthesis of phytotoxins. BGC-13 showed homology to the botcinic acid BGC from *B. cinerea* (82–84% identity). BGC-14 was found in two consecutive copies on the same scaffold and showed homology to the solanapyrone D BGC from *Alternaria solani* (49–51% identity). Two other T1PKS-like BGCs were identified in *M. fructicola* and *M. laxa* but not in *M. fructigena*: BGC-15 homologous to the brefeldin A BGC from *Penicillium brefeldianum* (49–51% identity), and BGC-16 homologous to the grayanic acid BGC from the lichen *Cladonia grayi* (33–37% identity). The PKS in BGC-17, which was identified only in *M. fructigena*, was homologous to the fumonisin BGC from *Fusarium oxysporum* (42% identity).

A single terpene biosynthetic gene cluster (BGC-18) was identified in the *Monilinia* genomes. It showed homology to the squalestatin S1 BGC in *Aspergillus* sp. (57–68% identity), which is an inhibitor of mammalian and fungal squalene synthases.

## Discussion

Extensive investigations were performed in this study to elucidate the diversity among the most important *Monilinia* species that induce brown rots on pome and stone fruit. The genomic resources available for *M. fructigena* (Landi et al., 2018), *M. fructicola* (De Miccolis Angelini et al., 2019), and *M. laxa* (Landi et al., 2020) were exploited to deepen the knowledge of evolutionary history and to identify genes associated with virulence, niche specialization and other phenotypic traits in the *Monilinia* genus.

As a first approach, the newly sequenced genomes of reference strains of the three *Monilinia* species were analyzed from a phylogenomic point of view. Although based on single fungal strains and a limited number of taxa, our genome-scale phylogeny clearly supports the genetic relationships among *M. fructicola*, *M. fructigena* and *M. laxa*, and representative species within the family *Sclerotiniaceae* as well as within the order *Helotiales* (Wijayawardene et al., 2018). The main phylogenetic groups defined by our analysis are congruent with the family clades recognized by Johnston et al. (2019). In agreement with Yin et al. (2015), *M. fructicola* proved genetically distant from *M. laxa* and *M. fructigena*, although the three species likely shared a common ancestor. According to previous studies, *Botrytis* and *Sclerotinia* were the closest related taxa to the *Monilinia* genus in the *Junctoriae* section, and all were in a monophyletic lineage strictly related to *Rustroemiaceae* (Holst-Jensen et al., 1997). The strict evolutionary relationship among the *Monilinia* genus, *B. cinerea* and *S. sclerotiorum* was shown for the first time by large-scale syntenic studies that in addition to conserved homologies, referred to relationships not only between genes but also between their genomic frameworks (Jiao and Schneeberger, 2020; Lichtin et al., 2020). High syntenic relationships were found among the three *Monilinia* genomes and, albeit minor, with *B. cinerea* and *S. sclerotiorum*. This further confirms the close evolutionary relationship of the genus with the other *Sclerotiniaceae* tested. The coding sequence divergence (measured by Ks values) is frequently used as a relative molecular clock to estimate divergence times from a common ancestor (Haug-Baltzell et al., 2015; Shi et al., 2019). Ks analysis among the three *Monilinia* genomes yielded results in agreement with the findings of phylogenomic studies, which confirmed that *M. laxa* and *M. fructigena* are phylogenetically closely related to each other, while *M. fructicola* is somewhat divergent. The three *Monilinia* genomes were genetically closer to those of *S. sclerotiorum* than *B. cinerea*, and *M. laxa* was the closest to the other fungi of the *Sclerotiniaceae* family that were tested.

Conserved syntenic blocks across the analyzed genomes were investigated for prediction of conservation of gene order and possible gene interactions (Walden et al., 2020). Pairwise comparisons revealed high proportions of genes with a syntenic depth of 1, even in the inter-genera comparisons, which indicated a low number of duplicated regions and a large prevalence of orthologous genes in the *Monilinia* genomes (Tang et al., 2015). Hence, gene duplication is not a driven process for diversity and adaptation, as it is in yeast and other fungi. Comparative synteny mapping among the *Monilinia*, *B. cinerea* and *S. sclerotiorum* genomes showed increasing numbers of small syntenic blocks in relation to evolutionary distance, probably due to genome rearrangements. Comparisons among the *Monilinia* genomes revealed the highest numbers of collinear syntenic genes per block, and hence the lowest numbers of total blocks, between the *M. fructicola* and *M. laxa* genomes. However, the large fragmentation of the *M. fructigena* genome assembly (131 scaffolds) as compared with *M. fructicola* and *M. laxa* (20, 49 scaffolds, respectively) might affect the results of the comparison. Nevertheless, the smaller fragmentation of syntenic blocks observed in the *M. fructigena/M. laxa* than in the *M. fructigena*/*M. fructicola* comparisons somewhat confirms the greater diversity of *M. fructicola* from the other *Monilinia* strains. In *M. fructigena*, a more frequent occurrence of syntenic breakpoints caused by insertion of non-syntenic genes linked to TE transposition was often detected. This suggests the occurrence of recombinational events due to TE activity in *M. fructigena*, as moving DNA into and out of a genomic region can cause gene transposition and local duplication, thus having a role in decreasing syntenic signals (Slot and Rokas, 2010; Wisecaver et al., 2014; Faino et al., 2016; Klein and O’Neill, 2018).

In the present work, the TEs content in the *Monilinia* genomes ranged from 6.0% to 7.1% of total size, and was comparable to those in the *B. cinerea* (5.1%) and *S. sclerotiorum* (6.6%) genomes. Previous investigations have reported 7% to 12% TEs in the *S. sclerotiorum* genome, which might have included some dubious annotations (Amselem et al., 2011, 2015; Derbyshire et al., 2017), and about 4% TEs in the *B. cinerea* genome (Porquier et al., 2016). These slight discrepancies might be due to the different annotation tools and pipelines used, as well as to the continuous updating of TE libraries. However, the present study confirmed the higher number of TEs in the *S. sclerotiorum* genome compared to *B. cinerea*, as well as a prevalence of DNA elements in the *S. sclerotiorum* genome (Santana et al., 2014) and of LTR elements in the *B. cinerea* genome (Amselem et al., 2015).

In the *Monilinia* genomes, Class I retrotransposons (especially of the LTR/Gypsy and LTR/Copia superfamilies) were the most abundant, as in most fungi (Muszewska et al., 2011; Santana et al., 2014; Donnart et al., 2017). However, while the LTR/Copia superfamily was in similar proportions in the three *Monilinia* genomes, LTR/Gypsy was much more abundant in *M. laxa* and contributed significantly to the high content of all LTRs in its genome. DNA transposons (represented mainly by DNA/Hat and DNA/Mariner superfamilies) were particularly abundant in *M. fructicola* and *S. sclerotiorum* (Santana et al., 2014; this study). The broad differences in TE types in the *Monilinia* genomes suggest that they have a significant role in evolution. Several TEs (e.g., LTR/Gypsy-2_PaPe-I or DNA/Mariner1_AO) were also common to the *B. cinerea* and *S. sclerotiorum* genomes, which suggest the same origin as in the *Monilinia*, although TEs likely expanded differently in the different genomes after the speciation process, as suggested by their diverse frequencies and cumulative lengths in the genomes analyzed here. Our results are hence suggestive of a different evolutionary role for TE proliferation events that occurred in the course of time in these genomes. The TE landscape in *M. laxa* and *M. fructicola*, indeed, showed an old activity burst of both LTR and DNA elements, with the current occurrence of inactive, degenerate TEs in both genomes. On the contrary, the TE landscape in *M. fructigena* suggests a more recent burst of active TEs that is probably still in place, which has resulted in a highly dynamic genome, and has probably caused marked genome reorganization (Kang et al., 2001; Zhou et al., 2007; Haas et al., 2009; Yoshida et al., 2016). Similarly, although to a lesser extent, the TE landscape in the *S. sclerotiorum* and *B. cinerea* genomes suggests their recent expansion events.

The older expansions of *M. laxa* and *M. fructicola* TEs compared to *M. fructigena* suggest that there might have been evolutionary pressure against them, to limit their activity in the genomes. Usually, the evolutionary pressure against inactive TEs is lower and their fragments can remain in genomic regions where active TEs are not allowed (Muszewska et al., 2019). In the *M. laxa* and *M. fructicola* genomes, we identified several conserved orthologous non-coding regions that harbored many non-autonomous TEs that maintained partial homology with genes associated with transposition in *M. fructigena*. TEs with structural features of functional domains that are necessary for autonomous transposition (Havecker et al., 2004; Casacuberta and González, 2013; Castanera et al., 2016) were detected only in *M. fructigena.* A similar result was observed in *S. sclerotiorum*, and to a much lesser extent, in *B. cinerea*, which suggests the presence of active TEs also in these genomes, in agreement with previous reports (Santana et al., 2014; Muszewska et al., 2019).

Microsynteny analysis of the *M. fructigena* genome revealed that synteny breaks were associated with the presence of active TEs, such as non-LTR/Tad1-2_ACap elements, including or associated with numerous RdDPs (∼300 annotated genes), which are involved in the dispersion of a variety of mobile elements, including fungal retroelements (Mathias et al., 1991). These findings are consistent with the recent burst of TEs, as mainly non-LTR elements, observed in *M. fructigena*. In addition, the high wobble-position GC content within RdDP suggests a possible horizontal transfer of these genes into the genome, as has been reported in bacteria (Tuller, 2011; Callens et al., 2021).

The investigation on the genome-wide occurrence of RIP in *Monilinia* showed that all the three strains showed RIP hallmarks, and that these are likely to be mediated by the RID and DIM-2-mediated DNA methylation pathways (Gladyshev, 2017), as suggested by detection of key genes of these pathways: the two methyltransferases (RID, DIM-2) and the DIM-2-associated cofactors. The only exception was the cofactor DIM-7, which was absent in the *Monilinia* genomes, in line with what was recently reported for *B. cinerea*, *S. sclerotiorum* and several other Ascomycetes (van Wyk et al., 2021). Slight differences among the three genomes were observed in terms of the extent of this phenomenon. *M. fructigena* showed a more intense transposition activity, linked to lower RIP index values and higher GC content, gene density and TE contents in RIP-affected regions, compared to *M. fructicola* and *M. laxa*. On the other hand, the *M. fructicola* genome showed a more active RIP system and the highest content of DNA transposons. This is consistent with observations carried out in other fungi that have shown that an active RIP system and other genome defense systems, such as RNAi-mediated mechanisms, are associated to high proportions of DNA TEs but few active TEs (Muszewska et al., 2017). We found very low, but still detectable, levels of RIP in the *B. cinerea* and *S. sclerotiorum* genomes. These findings agree with previous studies that have reported infrequent RIP mutations in both species (Derbyshire et al., 2017; Valero-Jiménez et al., 2019) and no evidence of RIP in selected TE sequences, such as DNA/Tc1-Mariner in *S. sclerotiorum* (Santana et al., 2014).

Repeat-induced point mutation processes operate during the sexual cycle and are exclusively available to fungi that are actively recombining (Selker, 1990; Gladyshev, 2017). The *Sclerotiniaceae* family include cosmopolitan broad host-range fungi that are subjected to different environmental conditions that might affect their reproduction modality (Aldrich-Wolfe et al., 2015). Regarding the *Monilinia* species, apothecia have been found rarely in the field for *M. fructigena* and *M. laxa* and have commonly been reported for *M. fructicola* (e.g., Holtz et al., 1998). Moreover, the use of classical and molecular markers for variation analysis indicates that sexual reproduction occurs in *M. fructicola* populations (Free et al., 1996; Michailides et al., 2007; Abate et al., 2018b). Overall, our results suggest different roles and occurrence frequencies of RIP in the three *Monilinia* genomes. The numerous active TEs and low frequency of RIP mutations in the *M. fructigena* genome would confirm the infrequent occurrence of the sexual process in its life cycle, in agreement with Martini and Mari (2014). On the contrary, the relatively high frequency of RIP mutations and few active TEs in *M. fructicola* would appear to be associated to frequent occurrence of sexual reproduction in this species (Holtz et al., 1998; Abate et al., 2018b).

The comparisons of the CAZyme repertoire among the *Monilinia*, *Botrytis* and *Sclerotinia* genomes revealed few differences in CAZyme classes and families, and in their substrates. As expected for pathogens that cause soft rot diseases, the pectin degrading glycoside hydrolases of family 28 were the most abundant CAZymes in the *Monilinia* genomes. Major differences were found in auxiliary activity enzymes of the AA3-2 subfamily, glycosyltransferases and some glycoside hydrolases. These included enzymes involved in basic polysaccharide degradation, such as mannosidases and glucosidases, and plant cell-wall-degrading enzymes, e.g., CAZymes involved in degradation of lignin and other polysaccharides, like arabinanes, which were detected in *M. laxa* and *M. fructicola* but were lacking in *M. fructigena*. These differences in plant cell-wall-degrading CAZymes might influence the pathogenicity of the *Monilinia* species and reflect differences in terms of the host plant and organ preferences known for the three pathogens. Although these *Monilinia* species can infect and cause disease on different plant species within the *Rosaceae* family, indeed, *M. fructigena* causes mainly fruit rot and less frequently twig blight and canker on pome fruit, and much more rarely on stone fruit, while *M. laxa* and *M. fructicola* cause blossom and twig blight as well as pre- and post-harvest fruit rot, where *M. fructicola* is more severe (Byrde and Willets, 1977; Martini and Mari, 2014; Papavasileiou et al., 2015).

Our whole-genome comparative analysis yielded a handful of candidate effectors common or specific to the three *Monilinia* strains. These included toxins and cell death elicitor proteins, such as: necrosis and ethylene-inducing like proteins (NLPs) orthologs to BcNEP1 of *B. cinerea* (Staats et al., 2007) and SsNEP1 of *S. sclerotiorum* (Guyon et al., 2014); cerato-platanins homologous to BcSpl1 and BcSpl2 of *B. cinerea* (Frías et al., 2011), which were already reported as candidate effectors in *M. fructicola* by Vilanova et al. (2021); a CVNH protein with a role in virulence and sclerotial development in *S. sclerotiorum* (Lyu et al., 2015); and a TNT and a heat-labile enterotoxin family detected in *M. laxa* and *M. fructigena*. The last enterotoxin family is typically related to human bacterial diseases, but also includes the Scabin toxin from *Streptomyces scabies* that has been implicated in plant pathogenesis (Lyons et al., 2016). Several other *Monilinia* candidate effectors have already been predicted in *S. sclerotiorum* (Derbyshire et al., 2017) and have shown homology to pathogenicity factors in other fungal species (Kulkarni et al., 2005), such as: a FKBP12 protein that significantly affects pathogenicity in *B. cinerea* (Gioti et al., 2006); proteins that display specific binding sites for chitin or cellulose or protease inhibitor activity, like a PEBP overexpressed in *M. fructicola* (De Miccolis Angelini et al., 2018); a HsbA protein that facilitates the binding between cutinase and hydrophobic surfaces and promotes cutin degradation (Ohtaki et al., 2006); and a fungal hydrophobin that has Bhp2 as an ortholog in *B. cinerea* (Mosbach et al., 2011). The potential role of candidate effectors in virulence and host adaptation in *Monilinia* species are worth further investigations.

Genome-based studies of several plant pathogenic fungi have shown that virulence-related genes involved in host specialization are frequently located in TE-rich genomic sequences or genomic compartments with higher rates of sequence evolution, such as accessory chromosomes, AT-rich regions, and clusters of tandem duplicated genes (Sánchez-Vallet et al., 2018; Valero-Jiménez et al., 2019). No clear correlations between the genome locations of TEs and putative virulence or pathogenicity factors were observed in the present study. The existence of a similar relationship remains elusive also for *S. sclerotiorum* (Derbyshire et al., 2017) and *B. cinerea* (Valero-Jiménez et al., 2019).

Overall, we detected 18 BGCs for secondary metabolites in the *Monilinia* genomes. Eleven orthologous gene clusters in the three *Monilinia* genomes coded for enzymes putatively responsible for production of siderophores, melanin, mycotoxins and bioactive compounds (*i.e.*, toxins, antibiotics, growth inhibitors). These might have important roles in their biology by functioning as genetic determinants of virulence (Steenwyk et al., 2020), by regulating interactions with the host plants, and by providing competitive fitness advantages in microbial interactions (Sass et al., 2019). From this perspective, these deserve further investigation. A PKS gene cluster with homology to a fumonisin BGC from *Fusarium oxysporum* (Proctor et al., 2008) was found only in *M. fructigena*, while six BGCs were common to both the *M. fructicola* and *M. laxa* genomes, but not to *M. fructigena*. These included BGCs for biosynthesis of inhibitors and antimicrobial substances, as well as BGCs for phytotoxins homologous to the well-known botcinic acid from *B. cinerea* (Dalmais et al., 2011) and solanapyrone D from *Alternaria solani* (Kim et al., 2015). BGC diversity in closely related fungal species might be due to horizontal gene transfer events (Campbell et al., 2012), chromosomal rearrangements, recruitment of genes from elsewhere in the genome followed by gene duplication, neofunctionalization and/or genome reorganization (Nützmann et al., 2016). Gene gains and losses might be associated with the presence of specific TEs adjacent to BGC regions (Tralamazza et al., 2019). TE activity in the *M. fructigena* genome can explain the absence of orthologs of BGCs in *M. fructicola* and *M. laxa.* For instance, active TEs in *M. fructigena* altered BGC-8, which includes a conserved module of *pks12*-*smr1* genes essential for sclerotial melanogenesis in *B. cinerea* (Schumacher, 2016; Zhou et al., 2017). Large TE insertions associated to inversion and translocation events led, indeed, to physical separation of the core *pks12* gene from the transcription-factor-encoding *smr1* gene, while the two genes were clustered together in both the *M. fructicola* and *M. laxa* genomes, as in *B. cinerea* and other *Leotiomycetes* studied (Schumacher, 2016). The three *Monilinia* species produce pigmented mature sclerotia, but differed for the pigmentation of their stromata and their colonies, which are typically buff, pale luteous or creamy yellow in *M. fructigena*, and grayish or hazel in *M. laxa* and *M. fructicola* (OEPP/EPPO, 2009; Petróczy et al., 2012) (see Figure 3). Therefore, it can be hypothesized that the TE alteration of BGC-8 affects melanogenesis in *M. fructigena*, with possible effects on the fungal biology (Cordero and Casadevall, 2017). However, this hypothesis and the role of BGCs in the *Monilinia* species remain to be clarified.

## Conclusion

Comparative genomic investigations were carried on new high-quality genome assemblies of strains of *M. fructicola*, *M. fructigena* and *M. laxa*, as important plant pathogens that cause heavy yield losses for pome and stone fruit worldwide. Phylogenetic investigations suggest that *M. laxa* and *M. fructigena* are close to each other, while *M. fructicola* is more distant from them, although they all derive from a common ancestor. The three *Monilinia* species are closely related to *B. cinerea* and *S. sclerotiorum*, within the family *Sclerotiniaceae*, and grouped in the “sclerotinioid” clade within *Helotiales.* These findings were confirmed by large-scale synteny analysis of the three *Monilinia* genomes and their strong syntenic relationship with *S. sclerotiorum* than *B. cinerea*. However, synteny results indicated that *M. laxa* is the closest to the other *Sclerotiniaceae* species investigated, which suggests that *M. laxa* was the earliest differentiated through the speciation process. The key divergences among the *Monilinia* genomes were seen by the diversities in type and abundance of TE sequences, as well as for the RIP hallmarks. TEs with functional domains were poorly present in the *M. fructicola* and *M. laxa* genomes. Active TEs were abundant in the *M. fructigena* genome and were often linked to functionally annotated genes associated with transposition activity. These features were also seen for the *S. sclerotiorum* genome, and to a lesser extent, for the *B. cinerea* genome. This suggests the occurrence during species evolution of an older TE burst in *M. laxa* and *M. fructicola*, compared to *M. fructigena*. These evolutionary differences were emphasized by active RIP mechanisms in *M. fructicola* and to a lesser extent in *M. laxa*, but not in *M. fructigena*, and they reflect differences in the occurrence of the sexual process in the three species. We identified several conserved BGCs for siderophores, mycotoxins, phytotoxins and other bioactive compounds in the three *Monilinia* genomes. Similarly, investigations of the CAZyme repertoires showed conserved enzyme families, presumably involved in pathogenicity, and the absence in *M. fructigena* of some enzymes involved in degradation of lignin and other polysaccharides, such as arabinans. This suggests distinctive features of *M. fructigena* that might have important outcomes on their pathogenicity. In *M. fructigena*, we observed a solid example of the impact of TE activity in the reshaping of the evolution of fungal genomes, to result in possible gene function alterations. Insertion of active TEs in BGC-8, which is involved in sclerotial melanogenesis in *B. cinerea* and is conserved in both *M. fructicola* and *M. laxa*, led indeed to a physical separation of the core biosynthetic *pks12* gene from the transcription-factor-encoding *smr1* gene. Furthermore, we identified several common or specific putative virulence factors, including toxins and cell-death-inducing proteins. No significant associations between secreted and effector-like proteins and TEs were found in the *Monilinia* genomes, which suggests that TE activity was not a dominant process in their evolution. The results obtained with the whole genome investigations here indicated that the three important pathogens that induce brown rot on pome and stone fruit, *M. fructicola*, *M. fructigena* and *M. laxa*, can be subjected to different, somewhat divergent, evolutionary pressures, probably due to their biology and adaptation to different environments and host plants. Although derived by an *in silico* analysis of single strains representative of each species, the genes of different functional categories identified in this study provide a solid basis for future investigations into the population biology of these important plant pathogens and on their interactions with host plants and associated microbial communities.

## Data Availability Statement

Publicly available datasets were analyzed in this study. This data can be found here: https://www.ncbi.nlm.nih.gov/assembly/GCA_008692225.1; https://www.ncbi.nlm.nih.gov/assembly/GCA_003260565.1; https://www.ncbi.nlm.nih.gov/assembly/GCA_009299455.1; https://www.ncbi.nlm.nih.gov/assembly/GCF_000143535.2; and https://www.ncbi.nlm.nih.gov/assembly/GCA_001857865.1.

## Author Contributions

RD and LL analyzed the data and wrote the first draft of the manuscript. CR analyzed part of the data and contributed to the writing. SP supervised the analyses and the writing. FF and GR supervised and complemented the writing and coordinated the collaboration of the authors. All authors read and approved the final manuscript and contributed to the study conception and design.

## Conflict of Interest

The authors declare that the research was conducted in the absence of any commercial or financial relationships that could be construed as a potential conflict of interest.

## Publisher’s Note

All claims expressed in this article are solely those of the authors and do not necessarily represent those of their affiliated organizations, or those of the publisher, the editors and the reviewers. Any product that may be evaluated in this article, or claim that may be made by its manufacturer, is not guaranteed or endorsed by the publisher.

## Footnotes

1
http://www.cabi.org/isc/datasheet/34747

2
https://genomevolution.org/coge

3
http://www.repeatmasker.org/RepeatModeler

4
https://www.girinst.org/censor

5
https://www.ncbi.nlm.nih.gov/orffinder

6
https://blast.ncbi.nlm.nih.gov/smartblast/smartBlast.cgi

7
www.ncbi.nlm.nih.gov

8
http://effectorp.csiro.au

9
http://antismash.secondarymetabolites.org

10
www.jcvi.org/smurf

11
https://fungiminions.shinyapps.io/FunGeneClusterS
