# Supplementary figures and images for: Tracking of Diversity and Evolution in the Brown Rot Fungi Monilinia fructicola, Monilinia fructigena, and Monilinia laxa
Source: Front Microbiol. 2022 Mar 9;13:854852. doi: 10.3389/fmicb.2022.854852 (PMC8959702; doi:10.3389/fmicb.2022.854852)

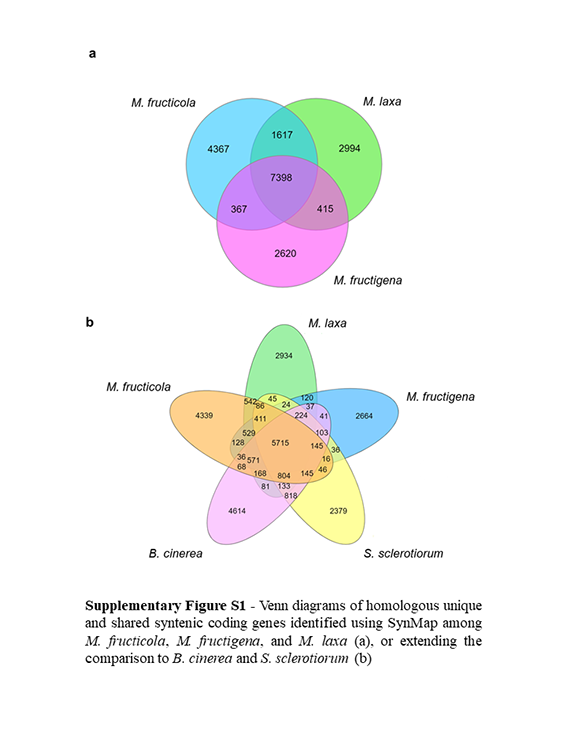

Supplement: Supplementary file 1 [file Image_1.tif]

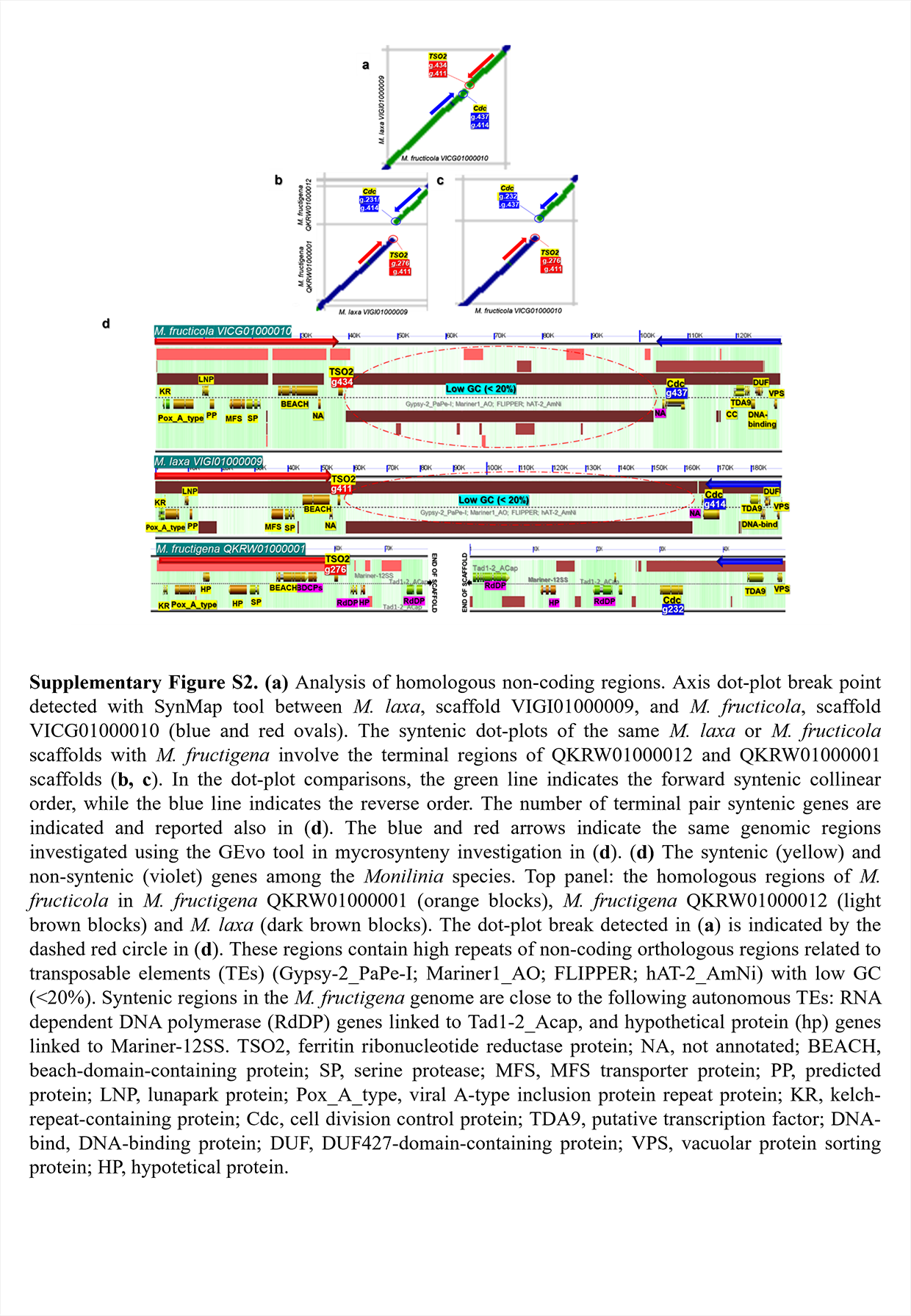

Supplement: Supplementary file 2 [file Image_2.tif]

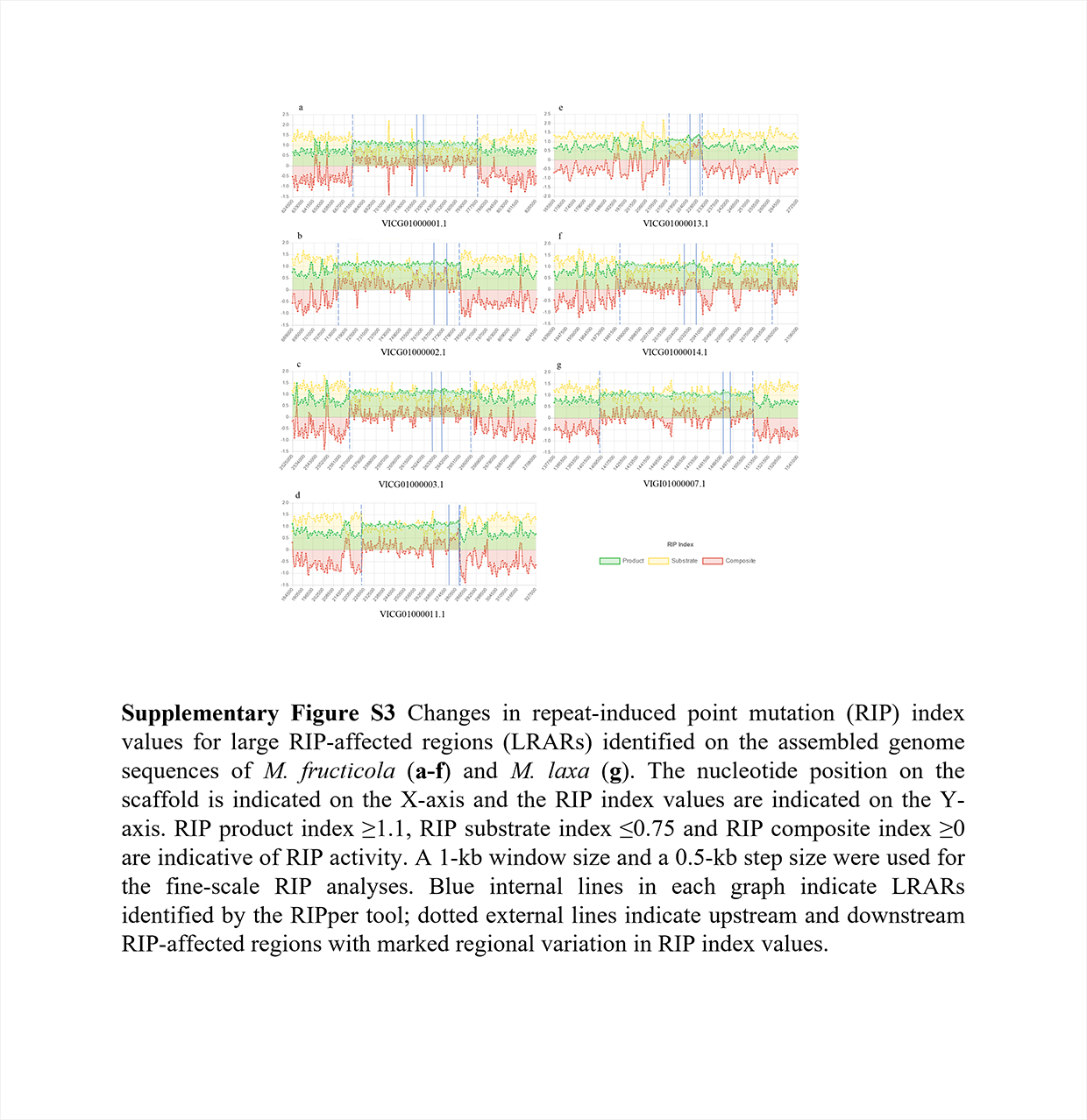

Supplement: Supplementary file 3 [file Image_3.tif]

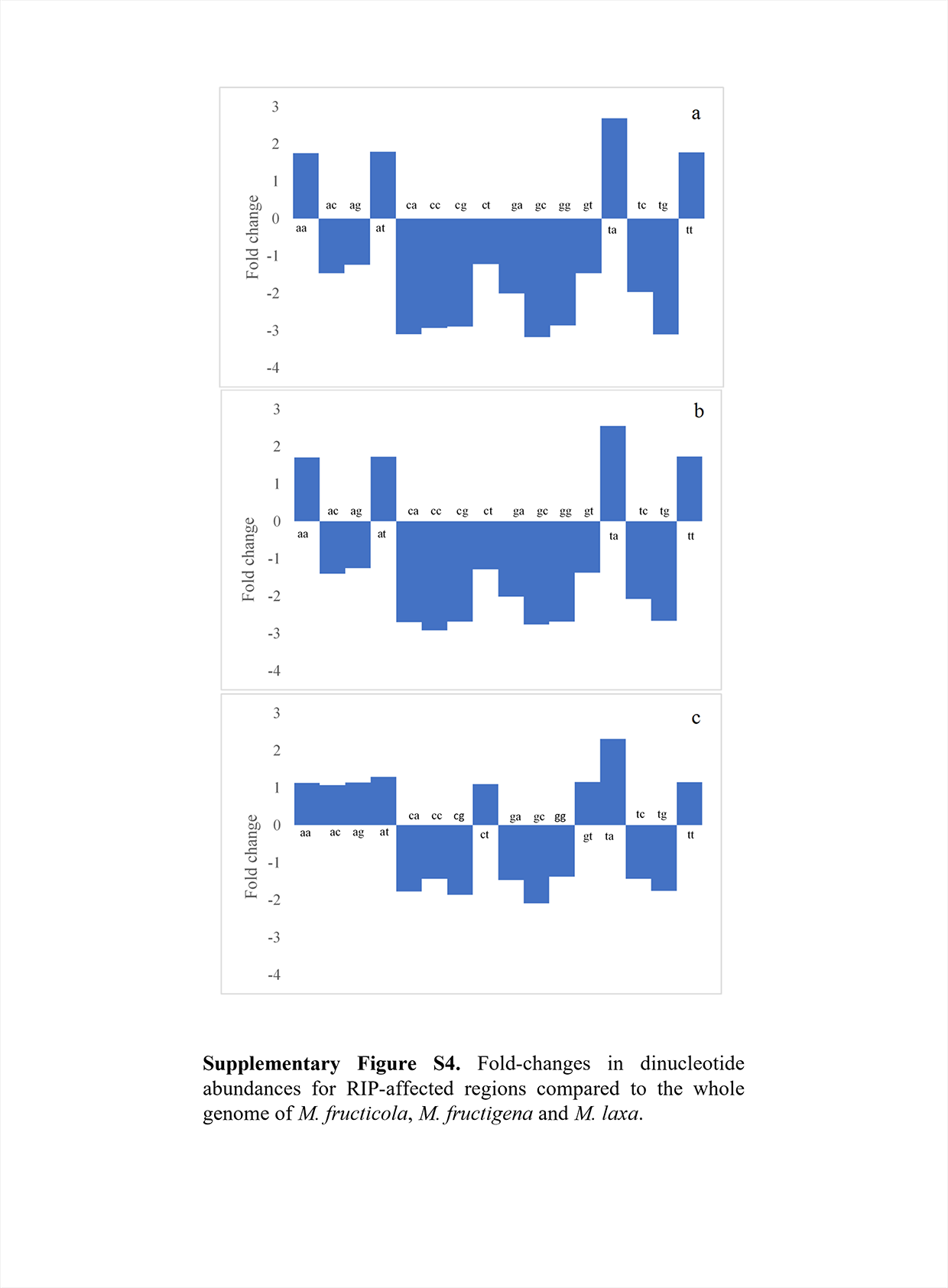

Supplement: Supplementary file 4 [file Image_4.tif]

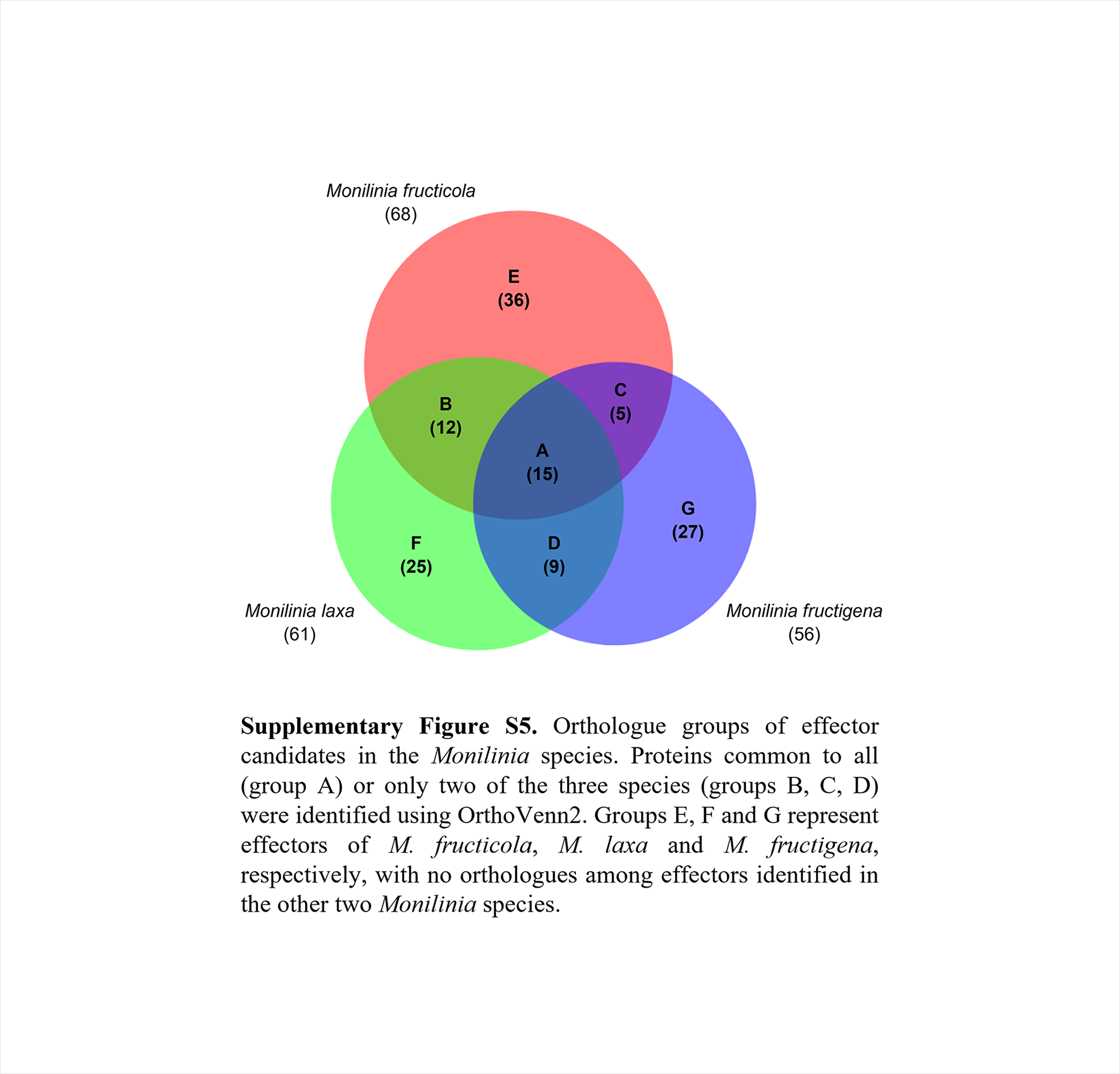

Supplement: Supplementary file 5 [file Image_5.tif]

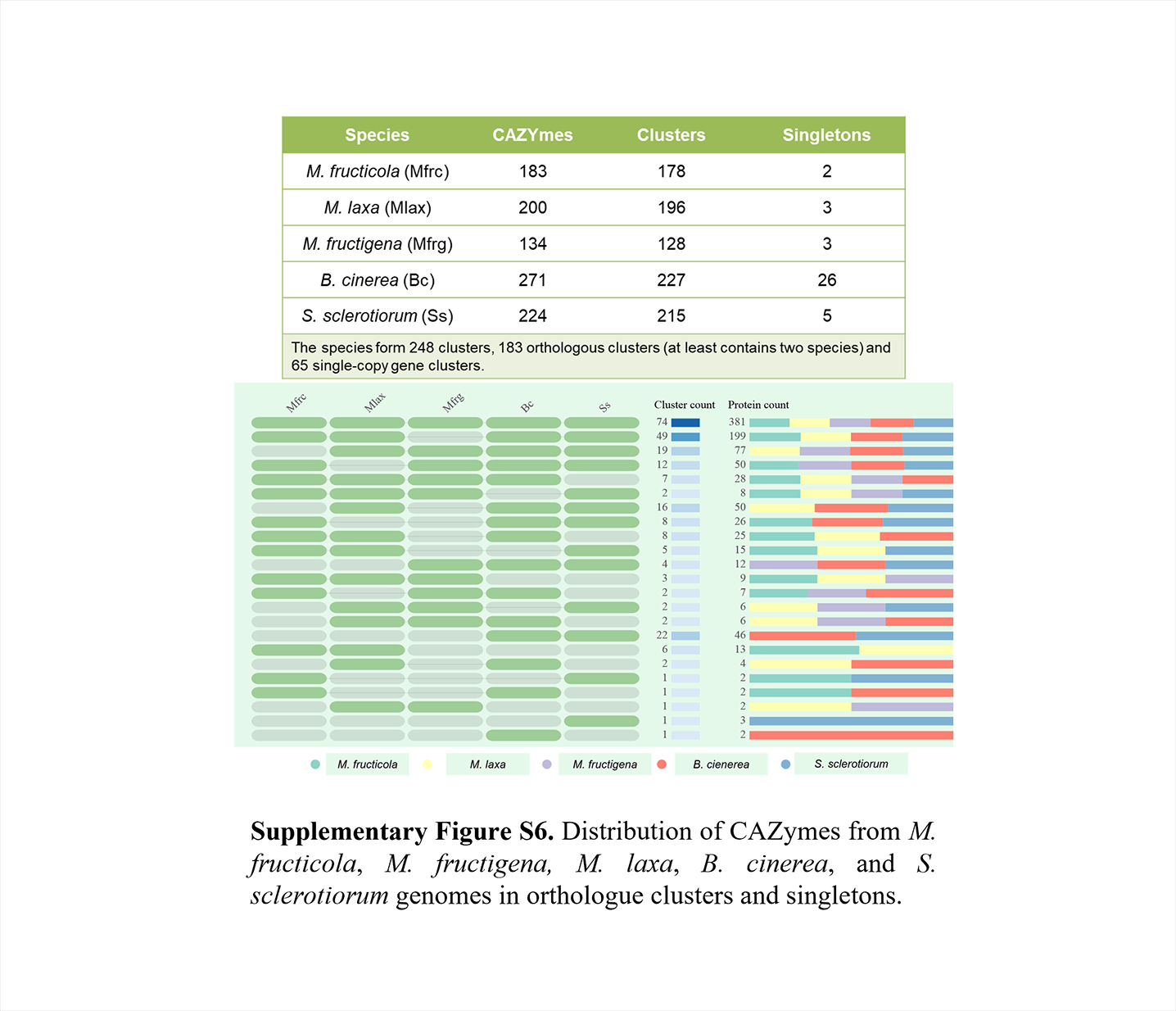

Supplement: Supplementary file 6 [file Image_6.tif]
