# Supplementary material for: Tracking of Diversity and Evolution in the Brown Rot Fungi Monilinia fructicola, Monilinia fructigena, and Monilinia laxa
Source: Front Microbiol. 2022 Mar 9;13:854852. doi: 10.3389/fmicb.2022.854852 (PMC8959702; doi:10.3389/fmicb.2022.854852)
Supplement: Supplementary file 7 [file Table_1.docx]

**Supplementary Table 1**. List of species within the order *Helotiales* submitted to phylogenetic analysis.

| **Species** | **Code/strain** | **Family^a^** | **Genbank accession number** | **Number of annotated proteins** |
| --- | --- | --- | --- | --- |
| *Botrytis cinerea* | B05.10 | *Sclerotiniaceae* | GCF_000143535.2 | 13,703 |
| *Cadophora* sp. | DSE1049 | *Ploettnerulaceae* | GCA_003073865.1 | 22,762 |
| *Chlorociboria aeruginascens* | IHIA39 | *Chlorociboriaceae* | GCA_002276475.2 | 8,648 |
| *Coleophoma cylindrospora* | BP6252 | *Dermateaceae* | GCA_003369665.1 | 14,177 |
| *Coleophoma crateriformis* | BP5796 | *Dermateaceae* | GCA_003369635.1 | 13,257 |
| *Diplocarpon rosae* | DortE4 | *Drepanopezizaceae* | GCA_002317995.1 | 13,761 |
| *Glarea lozoyensis* | ATCC 20868 | *Helotiaceae* | GCA_000409485.1 | 13,083 |
| *Hyaloscypha variabilis* | F | *Hyaloscyphaceae* | GCA_002865655.1 | 20,386 |
| *Marssonina brunnea* (=*Drepanopeziza brunnea*) | MB_m1 | *Drepanopezizaceae* | GCA_000298775.1 | 10,027 |
| *Marssonina coronariae* (=*Diplocarpon coronariae*) | NL1 | *Drepanopezizaceae* | GCA_002204255.1 | 9,355 |
| *Meliniomyces bicolor* (=*Hyaloscypha bicolor*) | E | *Hyaloscyphaceae* | GCA_002865645.1 | 18,604 |
| *Monilinia fructicola* | Mfrc123 | *Sclerotiniaceae* | GCA_008692225.1 | 13,749 |
| *Monilinia fructigena* | Mfrg269 | *Sclerotiniaceae* | GCA_003260565.1 | 10,800 |
| *Monilinia laxa* | Mlax316 | *Sclerotiniaceae* | GCA_009299455.1 | 12,424 |
| *Pezoloma ericae* | UAMH 7357 | *Discinellaceae* | GCA_002865625.1 | 16,783 |
| *Phialocephala scopiformis* | CBS 120377 | *Mollisiaceae* | GCA_001500285.1 | 18,567 |
| *Phialocephala subalpina* | UAMH 11012 | *Mollisiaceae* | GCA_900073065.1 | 20,173 |
| *Rhynchosporium agropyri* | 04CH-RAC-A.6.1 | *Ploettnerulaceae* | GCA_900074905.1 | 13,673 |
| *Rhynchosporium commune* | UK7 | *Ploettnerulaceae* | GCA_900074885.1 | 12,211 |
| *Rhynchosporium secalis* | 02CH4-6a.1 | *Ploettnerulaceae* | GCA_900074895.1 | 13,145 |
| *Rutstroemia* sp. | NJR-2017a BBW | *Rutstroemiaceae* | GCA_002946425.1 | 10,975 |
| *Sclerotinia sclerotiorum* | 1980 UF-70 | *Sclerotiniaceae* | GCA_001857865.1 | 11,130 |
| *Sclerotinia borealis* | F-4128 | *Sclerotiniaceae* | GCA_000503235.1 | 10,166 |

^a^According to the CABI database (<http://www.speciesfungorum.org/Names/fundic.asp>)
