# Supplementary material for: Tracking of Diversity and Evolution in the Brown Rot Fungi Monilinia fructicola, Monilinia fructigena, and Monilinia laxa
Source: Front Microbiol. 2022 Mar 9;13:854852. doi: 10.3389/fmicb.2022.854852 (PMC8959702; doi:10.3389/fmicb.2022.854852)
Supplement: Supplementary file 14 [file Table_8.docx]

**Supplementary Table 8.** Orthologous groups of putative biosynthetic gene clusters (BGCs) in the genomes of *M. fructicola* (MFRC), *M. laxa* (MLAX) and *M. fructigena* (MFRG).

| **Type** | **BGC^1^** | ***Monilinia*** | **Genomic location** | **Size** | **Number** | **Core gene** | | **Homologous gene clusters in MIBiG^2^** | | |
| --- | --- | --- | --- | --- | --- | --- | --- | --- | --- | --- |
|  | **code** | **species** | **on scaffold** | **(bp)** | **of genes** | **Code** | **Description** | **Compound and species (accession)** | **Identity (%)** | **E-value** |
| NRPS | 1 | MFRC ^(A,S)^ | VICG01000001.1 (1,543,288...1,588,042) | 44,755 | 13 | EYC84_006441 | Non-ribosomal siderophore peptide synthase Sid2 | Ferrichrome from *Aspergillus oryzae* (BGC0000900) | 64 | 0.0 |
|  |  | MFRG ^(S)^ | QKRW01000024.1 (115,327... 142,257) | 26,931 | 7^3^ | DID88_004976 |  |  | 68 | 0.0 |
|  |  | MLAX ^(S)^ | VIGI01000003.1 (2,594,978... 2,606,571) | 11,594 | 2 | EYC80_00619 |  |  | 75 | 0.0 |
|  | 2 | MFRC ^(S)^ | VICG01000003.1 (805,365... 841,369) | 36,005 | 9 | EYC84_005166 | Non-ribosomal peptide synthetase | Aspercryptins from *Aspergillus nidulans* FGSC A4 (BGC0001515) | 32 | 0.0 |
|  |  | MFRG ^(A,S)^ | QKRW01000030.1 (238,176... 281,592) | 43,417 | 10^3^ | DID88_009596 |  |  | 34 | 0.0 |
|  |  | MLAX ^(S)^ | VIGI01000002.1 (2,431,868... 2,467,714) | 35,847 | 10 | EYC80_005178 |  |  | 34 | 0.0 |
|  | 3 | MFRC ^(S)^ | VICG01000001.1 (273,679... 286,890) | 13,212 | 3 | EYC84_006108 | Non-ribosomal peptide synthetase dtxS1 | Destruxin A from *Metarhizium robertsii* ARSEF 23 (BGC0000337) | 39 | 0.0 |
|  |  | MLAX ^(S)^ | VIGI01000017.1 (264,203… 273,250) | 9,048 | 3 | EYC80_010849 |  |  | 39 | 0.0 |
|  | 4 | MFRC ^(A,S)^ | VICG01000006.1 (1,551,859... 1,596,637) | 44,779 | 14^3^ | EYC84_000428 | Non-ribosomal peptide synthetase | Aspirochlorine from *Aspergillus oryzae* RIB40 (BGC0001123) | 42 | 0.0 |
|  |  | MFRG ^(A,S)^ | QKRW01000016.1 (257,782... 302,825) | 45,044 | 15^3^ | DID88_003156 |  |  | 42 | 0.0 |
|  |  | MLAX ^(A,S)^ | VIGI01000007.1 (1,873,073... 1,916,168) | 43,096 | 13^3^ | EYC80_001899 |  |  | 44 | 0.0 |
| NRPS-like | 5 | MFRC ^(A)^ | VICG01000003.1 (1,745,760... 1,786,134) | 40,375 | 12 | EYC84_005495 | NRPS-like enzyme | Fusaric acid from *Fusarium verticillioides* 7600 (BGC0001190) | 34 | 0.0 |
|  |  | MFRG ^(A)^ | QKRW01000007.1 (182,083... 225,333) | 43,251 | 13 | DID88_005839 |  |  | 34 | 0.0 |
|  |  | MLAX ^(A)^ | VIGI01000002.1 (1,523,635... 1,566,884) | 43,250 | 16 | EYC80_004841 |  |  | 34 | 1e-126 |
|  | 6 | MFRC ^(A)^ | VICG01000003.1 (3,469,820... 3,512,427) | 42,608 | 15 | EYC84_006020 | L-aminoadipate-semialdehyde dehydrogenase large subunit protein | Myxalamid from *Stigmatella aurantiaca* (BGC0001022) | 36 | 1e-34 |
|  |  | MFRG ^(A)^ | QKRW01000014.1 (99,969... 142,580) | 43,612 | 14 | DID88_001835 |  |  | 29 | 4e-133 |
|  |  | MLAX ^(A)^ | VIGI01000002.1 (142,713... 172,817) | 30,105 | 9 | EYC80_004501 |  |  | 29 | 2e-96 |
|  | 7 | MFRC ^(A,S)^ | VICG01000004.1 (532,539... 578,877) | 46,339 | 12 | EYC84_002996 | NRPS-like enzyme | Fusarielin H from *Fusarium graminearum* (BGC0001600) | 28 | 6e-116 |
|  |  | MFRG ^(A)^ | QKRW01000003.1 (207,279... 250,681) | 43,402 | 11 | DID88_008175 |  |  | 32 | 2e-158 |
|  |  | MLAX ^(S)^ | VIGI01000005.1 (2,612,778... 2,646,224) | 33,447 | 12 | EYC80_000712 |  |  | 25 | 9e-59 |
| T1PKS | 8 | MFRC ^(A,S)^ | VICG01000012.1 (1,593,612... 1,638,095) | 44,484 | 13^3^ | EYC84_009053 | Polyketide synthase pks12 | 1,3,6,8-Tetrahydroxynaphthalene from *Glarea lozoyensis* (BGC0001258) | 63 | 0.0 |
|  |  | MLAX ^(A,S)^ | VIGI01000012.1 (589,110... 633,850) | 44,741 | 8^3^ | EYC80_007403 |  |  | 62 | 0.0 |
|  | 9 | MFRC ^(A)^ | VICG01000004.1 (3,155,388... 3,224,542) | 69,155 | 20 | EYC84_003814 | Polyketide synthase  pks13 | Melanin from *Bipolaris oryzae* (BGC0001265) | 36 | 4e-08 |
|  |  | MFRG ^(A)^ | VIGI01000049.1 (138,492... 184,083) | 45,592 | 10 | DID88_000324 |  |  | 48 | 0.0 |
|  |  | MLAX ^(A)^ | VIGI01000019.1 (47,2161... 51,8121) | 45,961 | 12 | EYC80_010372 |  |  | 47 | 0.0 |
|  | 10 | MFRC ^(A,S)^ | VICG01000002.1 (2,015,892...2,061,522) | 45,631 | 22^3^ | EYC84_004443 | Atrochrysone carboxylic  acid synthase | Neosartorin from *Aspergillus novofumigatus* IBT 16806 (BGC0001988) | 67 | 0.0 |
|  |  | MFRG ^(A,S)^ | QKRW01000002.1 (1,055,796...1,100,384) | 44,589 | 17 | DID88_008775 |  |  | 67 | 0.0 |
|  |  | MLAX ^(A,S)^ | VIGI01000001.1 (1,661,517... 1,706,305) | 44,789 | 20 | EYC80_003965 |  |  | 67 | 0.0 |
|  | 11 | MFRC ^(A)^ | VICG01000013.1 (101,820... 149,654) | 47,835 | 15 | EYC84_009316 | Polyketide synthase | Neurosporin A from *Neurospora crassa* OR74A (BGC0001697) | 41 | 0.0 |
|  |  | MFRG ^(S)^ | QKRW01000023.1 (553,027... 577,912) | 24,886 | 10 | DID88_004554 |  |  | 44 | 0.0 |
|  |  | MLAX ^(A,S)^ | VIGI01000011.1 (135,171... 171,689) | 36,519 | 12^3^ | EYC80_009229 |  |  | 47 | 7e-154 |
|  | 12 | MFRC ^(S)^ | VICG01000003.1 (1,815,001... 1,830,595) | 15,595 | 3 | EYC84_005518 | Polyketide synthase | Cornexistin from *Paecilomyces divaricatus* (BGC0001557) | 34 | 4e-122 |
|  |  | MFRG ^(S)^ | QKRW01000007.1 (256,736... 270,963) | 14,228 | 2 | DID88_005852 |  |  | 42 | 0.0 |
|  |  | MLAX ^(A,S)^ | VIGI01000002.1 (1,430,942... 1,492,620) | 61,679 | 18^3^ | EYC80_004822 |  |  | 42 | 0.0 |
|  | 13 | MFRC ^(A,S)^ | VICG01000007.1 (112,758... 160,174) | 47,417 | 13^3^ | EYC84_002172 | Polyketide synthase | Botcinic acid from *Botrytis cinerea* B05.10 (BGC0001892) | 84 | 0.0 |
|  |  | MLAX ^(A,S)^ | VIGI01000006.1 (197,543... 258,358) | 60,816 | 15^3^ | EYC80_000788 |  |  | 82 | 0.0 |
|  | 14 | MFRC ^(A,S)^ | VICG01000011.1 (1,548,336... 1,617,212) | 68,877 | 23^3^ | EYC84_010277 | Polyketide synthase | Solanapyrone D from *Alternaria solani* (BGC0000146) | 49 | 0.0 |
|  |  |  | VICG01000011.1 (2,068,052... 2,115,429) | 47,378 | 11 | EYC84_010432 |  |  | 49 | 0.0 |
|  |  | MLAX ^(A,S)^ | VIGI01000015.1 (226,455... 270,586) | 44,132 | 8^3^ | EYC80_009800 |  |  | 51 | 0.0 |
|  |  |  | VIGI01000015.1 (656,257... 724,660) | 68,404 | 16^3^ | EYC80_009916 |  |  | 51 | 0.0 |
|  | 15 | MFRC ^(A,S)^ | VICG01000013.1 (346,324... 444,776) | 98,453 | 26 | EYC84_009381 | Polyketide synthase | 4-Epi-15-epi-brefeldin A from *Penicillium brefeldianum* (BGC0001141) | 43 | 0.0 |
|  |  | MLAX ^(A,S)^ | VIGI01000011.1 (407,913... 454,473) | 46,561 | 9^3^ | EYC80_009297 |  |  | 43 | 0.0 |
|  | 16 | MFRC ^(A)^ | VICG01000008.1 (1,843,971... 1,887,114) | 43,144 | 16 | EYC84_001177 | Non-reducing polyketide synthase | Grayanic acid from *Cladonia grayi* (BGC0001266) | 37 | 1e-136 |
|  |  | MLAX ^(S)^ | VIGI01000010.1 (1,733,241...1,753,245) | 20,005 | 6 | EYC80_007135 |  |  | 33 | 1e-55 |
|  | 17 | MFRG ^(A)^ | QKRW01000015.1 (477,664... 520,191) | 42,528 | 7 | DID88_002133 | Polyketide synthase | Fumonisin from *Fusarium oxysporum* (BGC0000063) | 42 | 0.0 |
| Terpene | 18 | MFRC ^(A)^ | VICG01000007.1 (1,071,650... 1,093,266) | 21,617 | 9^3^ | EYC84_002412 | Squalene synthetase | Squalestatin S1 from *Aspergillus* sp. Z5 (BGC0001839) | 68 | 0.0 |
|  |  | MFRG ^(A)^ | QKRW01000028.1 (422,201... 442,920) | 20,720 | 7 | DID88_002933 |  |  | 60 | 7e-175 |
|  |  | MLAX ^(S)^ | VIGI01000006.1 (1,176,533... 1,188,353) | 11,821 | 6 | EYC80_001027 |  |  | 57 | 2e-142 |

^1^BGCs identified by antiSMASH fungal version 5.2.0 ^(A)^ and/or SMURF ^(S)^ on the *Monilinia* genomes

^2^Similarity of the core gene in the *Monilinia* BGCs to homologous genes within BGCs in the repository of known biosynthetic gene clusters MIBiG (The Minimum Information about a Biosynthetic Gene cluster).

^3^Co-regulated genes detected by using transcriptome data from germinating conidia and actively growing mycelia (De Miccolis Angelini et al. 2018), the genome sequences (this work) and the prediction tool for fungal gene clusters FunGeneClusterS (Andersen et al., 2013).
